# The Unequal Effects of the COVID-19 Pandemic on Political Interest Representation

**DOI:** 10.1007/s11109-022-09842-x

**Published:** 2022-12-31

**Authors:** Gregory Eady, Anne Rasmussen

**Affiliations:** 1 Department of Political Science and the Center for Social Data Science (SODAS), University of Copenhagen, Øster Farimagsgade 5, 1153 Copenhagen K, Denmark; 2 Department of Political Science, University of Copenhagen, Øster Farimagsgade 5, 1153 Copenhagen K, Denmark; 3 Department of Political Economy, King’s College, London, UK; 4 Department of Comparative Politics, University of Bergen, Bergen, Norway

**Keywords:** Political representation, Political behavior, Interest groups, Crisis, Covid-19, Corona, Organized interests

## Abstract

The COVID-19 pandemic is viewed by many as the biggest global crisis since WWII and had profound effects on the daily lives of people and decision-making worldwide. Using the pandemic as a system-wide agenda shock, we employ a difference-in-differences design to estimate its causal effects on inequalities in political access, and social media prominence among business interests and NGOs. Our argument is twofold. First, the urgency and uncertainty of crises incentivized decision-makers to privilege providing access to business groups over securing inclusivity in the types of interests consulted. Second, NGOs compensated by increasing prominence in public communications. Our analysis of data from over 10,000 interest groups from over 100 countries registered in the European Union supports these hypotheses. Business interests successfully capitalized on the crisis in insider access, while NGOs increased prominence on social media. The results have wider implications for understanding how large-scale crises affect inequalities in representation.

**Supplementary Information:**

The online version contains supplementary material available at 10.1007/s11109-022-09842-x.

The COVID-19 Pandemic has been viewed as the biggest crisis since World War II and has threatened the earnings of a wide range of industries and profoundly affected the daily lives of citizens. This has led a broad range of interest groups to mobilize, and governments to actively reach out for their expertise. Although the crisis reduced the options for face-to-face lobbying, substantial amounts of lobbying has occurred during the pandemic. In the US, for instance, substantial lobbying was directed toward the $2.2 trillion Coronavirus Aid, Relief, and Economic Security (CARES) Act—the largest aid package in US history.^1^ In Europe, lobbying efforts were, for example, heavily directed toward the €750 billion extraordinary European Union (EU) Recovery Effort.^2^

Lobbying during COVID-19 is widely criticized for its lack of transparency and potential to reinforce existing inequalities in interest representation. In line with Churchill’s famous words to “never let a good crisis go to waste,” business interests have been accused of exploiting the crisis to their own benefit.^3^ Business stakeholders have, for instance, lobbied for less stringent environmental regulations to combat the financial challenges of the crisis.^4^ They have been criticized for reinventing old demands or formulating new ones relying on the crisis to justify their claims (Winfield, 2020).

The threats and uncertainty engendered by a crisis—and the resulting urgency of policy responses (Boin et al., 2006)—have the potential to greatly affect representation by changing how much decision-makers prioritize obtaining advice from different types of interest groups. Such changes are important due to their potential to contribute to inequality in political representation with decision-makers favoring some segments of citizens and types of organized interests over others (Gilens & Page, 2014). Yet, despite speculation about the winners and losers of COVID-19, we lack systematic research into the effects of the pandemic—and crises in general—on inequalities in interest representation.

Our aim is therefore to clarify the theoretical and empirical connections between crisis and inequality in interest representation by using the onset of the COVID-19 pandemic as a pan-societal shock to the political agenda. The pandemic provides a unique opportunity to examine these connections due to its wide-ranging impacts across broad sets of different types of interests. We document the effects of the COVID-19 pandemic on differences between NGOs and business interests in both (1) political access, and (2) public communications during the first crucial months of the pandemic. To do so, we use novel panel data of meetings with the European Commission and the social media (Twitter) activity of 11,967 interest groups from 116 countries included in the EU Transparency Register. Our theoretical expectation is that the COVID-19 crisis had differential effects on the relative prominence of business interests versus NGOs in access to policy-makers and social media usage. We argue that the threat, uncertainty, and urgency of the crisis increased the need for policy-makers to obtain expert information from and grant access to business groups to produce fast and effective outputs protecting health, public safety, and firm survival. By contrast, we expect that the crisis lowered the priority decision-makers attached to securing input from broader societal interests, which contributed to increasing pressure on NGOs to expand their prominence in public communication (e.g. social media) to reach and mobilize their broader and more diffuse constituencies.

Our empirical analysis focuses on the input side of policy-making and finds broad support for these expectations. The COVID-19 crisis substantially increased political access to EU policy-makers among business interests relative to NGOs, whereas NGOs increased their relative prominence on social media. Furthermore, we use text data concerning the content of each meeting and social media post to provide further evidence that the results are driven by the pandemic. Finally, we show that the results are not simply due to differences in interest groups’ existing access to economic resources.

Our findings show that crises and abrupt agenda changes can cause substantial changes in the prominence of different types of interest groups, but that the effects can vary between different channels of activity. They serve as an important stepping stone for further analysis of the implications of crisis for interest representation, with broad consequences for political governance and democratic legitimacy.

## Theoretical Framework

### Crisis and Inequalities in Interest Representation

According to Boin et al. (2006), a crisis is defined by the presence of three characteristics. First, it involves a threat to the core values of a society, such as safety and security or welfare and health. Second, it induces a sense of urgency: the need to act fast. Third, it is characterized by uncertainty both in the nature of the crisis and actions to tackle it. The COVID-19 pandemic captures all three elements: it represents a pan-societal threat to a number of values, with strong pressure on policy-makers to act in an environment with considerable uncertainty about how best to tackle the crisis. From the literature, we know that crises can lead to dramatic and radical shifts of political agendas. We know less, however, about how they affect interest representation in general (Birkland, 1998; Furnas et al., 2021) and inequalities in interest group access in particular. An important recent exception is Junk et al. (2021), which investigates the drivers behind how groups perceive changes in their political access as a result of the pandemic. They find that, even when controlling for affectedness, business interests were more likely than societal interests to rate their access as having increased (see also Crepaz et al., 2022). Our study complements these findings by theorizing and investigating how the pandemic might have differentially impacted patterns of interest representation in the political and public arena using behavioral data on (a) political access to meetings with policy-makers and (b) public communication on social media activity before and during the pandemic.

By “access” we refer to a two-way interaction, in which organized interests use insider strategies to approach policy-makers who grant them the opportunity to be heard (Bouwen, 2004; Eising, 2007; Junk, 2019, Rasmussen & Gross, 2015). It is frequently viewed as an important step to gaining actual political influence. According to a Washington saying, “If you’re not at the table, you’re on the menu” (Schlozman et al., 2012, p. 309). By “public communication”, we refer to social media activity used by groups. It can serve a broad range of purposes, including (but not restricted to) outsider lobbying (Kollman, 1998; der Graaf et al., 2016), where organized interests aim to generate attention and support by appealing to broader constituencies and the wider public (including citizens and other interest groups).

For each arena, we focus on potential shifts in the prominence of business interests versus NGOs before and during the pandemic. This allows us to contrast how activities of groups representing specific economic interests with those representing (broader) societal ones (Flöthe & Rasmussen, 2019; Gilens & Page, 2014; Schattschneider, 1960) are affected by the pandemic. By “business interests” we refer to both firms and business associations. By “NGOs”, we refer to organizations that represent societal and identity interests as opposed to economic interests. Some NGOs are involved in providing diffuse public goods (e.g., environmental and consumer groups), whereas others promote the views of specific identity subgroups (e.g., LGBT support groups, women’s associations, or particular hobbies).^5^

Regardless of whether one looks at insider access to policy-makers or the public communications of groups on social media, one challenge in assessing potential inequalities between different group types in these tools is that there is typically no established benchmark for judging what the alternative to a given pattern of interest representation looks like (e.g. Lowery et al., 2015, Schlozman et al., 2012). While scholars often rely on raw counts of different types of substantive interests, they recognize that balanced systems may not simply be those with equal levels of activity from different group types, because some groups may constitute a larger share of certain interest group populations to begin with (e.g. Gray & Lowery, 2000, Rasmussen & Carroll, 2014, Schattschneider, 1960, Schlozman et al., 2012).

Rather than departing from a specific benchline, we examine the prominence of NGOs versus business interests in both access to policy-makers and social media activity by investigating the relative changes in access for these two groups over time. This acknowledges that crises have the potential to either magnify or minimize their prominence relative to the pre-crisis status quo.

### Crisis as a Disturbance to Societal Interests

In the terminology of pluralist interest group theory, crisis can be viewed as a disturbance in society (e.g. Bentley, 1908, Truman, 1951). In the words of Truman (1951, p. 511), a “disturbance in established relationships anywhere in society may produce new patterns of interaction aiming at restricting or eliminating the disturbance.” According to this logic, the shifts in the agenda caused by a crisis can be seen as having the potential to affect patterns of interest representation: interests with large stakes might intensify lobbying efforts whereas other types of stakeholders whose interests are less disturbed might scale their efforts down. In the case of the COVID-19 crisis, these disturbances were wide-ranging and cut across social, political, economic, demographic, and cultural divides. This provides us with a unique opportunity to clarify the empirical connection between crisis and inequality in interest representation.

At the same time, disturbance theory alone is not sufficient for understanding the impact of the COVID-19 pandemic on patterns of interest representation. While some specific economic sectors might be more affected by the pandemic than others, the pan-societal nature of COVID-19 makes it difficult to establish a priori that either business interests or NGOs as a whole should be more disturbed and incentivized to mobilize. According to a recent study by Furnas et al. (2021), “in effect, the pandemic acts as a shock disrupting virtually every sector, thus putting each in plausible need of legislative ’champions”’ (Furnas et al., 2021, p. 24). Moreover, while disturbances might act as a demand mechanism that stimulates interest groups to mobilize, the relative prominence of different types of groups also depends on whether policy-makers supply them with access. In the following section, we therefore theorize how crisis can be expected to affect how decision-makers prioritize the allocation of access to different types of groups.

### How Crisis Affects Inequalities in Access to Policy-Makers

Our argument is that the characteristics of crisis affect how policy-makers weigh the importance of different types of resources, which may ultimately affect the ability of different types of interests to gain access to policy-makers. To develop this argument, we conceptualize lobbying as an exchange relationship in which policy-makers grant interest groups access in exchange for various kinds of goods that groups supply, e.g. technical expertise, financial contributions, and public standing (Broscheid & Coen, 2007; Rasmussen et al., 2021, Witko, 2006). By supplying these goods, interest groups in principle help policy-makers increase both input and output legitimacy (e.g. Scharpf, 2009). Input legitimacy concerns ensuring public support for, and participation in, policy-making. It can, for example, be strengthened by interest groups transmitting political information about citizens and stakeholders to policy-makers (De Bruycker & Rasmussen, 2021). Output legitimacy, on the other hand, concerns ensuring that policy-makers adopt high quality outputs to solve the challenges at hand. Interest groups can strengthen this by supplying technical expertise to policy-makers who do not necessarily have the resources to specialize in all areas (Bouwen, 2004).

The characteristics of crisis—urgency, threat, and uncertainty—can be expected to lead policy-makers to prioritize boosting output legitimacy over input legitimacy. This lower prioritization of input legitimacy in the early phase of the pandemic is something that has been observed in the broader literature on the democratic lessons of the COVID-19 response. In Bekker et al.’s (2020) survey of 48 countries, almost three-quarters of the countries examined centralized and concentrated decision-making during the early phase of the pandemic. According to them, “minority needs and impact are easily overlooked as democratic policy deliberation (a policy’s ‘input legitimacy’) is temporarily postponed or even shut down altogether” (Bekker et al., 2020, p. 854).

Hence, faced with strong time pressures and obligations to provide solutions to protect health, public safety, or prop up financially struggling businesses, policy-makers are predicted to place higher emphasis on interacting with interest groups that can provide technical input to work out fast solutions to a crisis. Similarly, the high uncertainty involved in many of the policy choices that policy-makers need to make is likely to increase the value of obtaining expertise during crisis compared to periods of ordinary policy-making. Furthermore, decision-makers may be willing to de-prioritize consulting and obtaining input from a broad range of societal interests during the exceptional circumstances of a crisis where social acceptance of policies matters less. The uncertainty and threats introduced by the crisis can thus justify and provide the political flexibility to shift priorities and provide access to a less societally representative set of stakeholders.

Such a prioritization of output over input legitimacy can be expected to benefit business interests, typically valued for their technical expertise compared to other interest groups (e.g. Coen, 2007, Dür & Mateo, 2013, but see De Bruycker, 2016). Business interests represent concentrated constituencies making it easier for them to acquire staff and resources to invest in and build up technical and informational capacity. Business interests are also more likely than other types of interest groups to hire revolvers with expertise and polfitical connections (Baumgartner et al., 2009; Strickland, 2020). Because of their relatively high knowledge of the scientific and political details of their policy sectors, business interests are therefore typically seen as attractive partners for policy-makers. Not surprisingly, they have therefore been shown to enjoy a comparative advantage over NGOs when it comes to using insider strategies and obtaining access (e.g. Coen, 2007, Dür & Mateo, 2013).

By contrast, NGOs are often viewed as structurally disadvantaged in obtaining political access. As an example, Mahoney (2004, p. 505) explains: “some types of groups are generally better endowed financially (i.e. the business groups) than others. Therefore, trade, professional and cross-sectoral business groups should be expected to have more income at their disposal than citizen or culture groups and thus be likely to have a higher probability of being included in the committee system.” Instead, NGOs are often perceived as being particularly important for receiving political information about the positions of citizens in order to boost the input legitimacy of policy-making (e.g. Mahoney & Beckstrand, 2011), which can be expected to matter less during crisis.

In sum, we expect that the high focus on increasing output legitimacy to respond quickly to the uncertainty of the pandemic benefited business groups relative to NGOs with respect to obtaining access to policy-makers.

#### Hypothesis 1

The COVID-19 pandemic increased political access for business interests relative to NGOs.

### How Crisis Affects Inequalities in Social Media Activity

NGOs can be expected to respond to crisis by increasing their prominence in other channels of activity through, for instance, public communications on social media. Not all activity on social media is directed at lobbying, but it is one option for groups to pursue outsider lobbying, i.e. attempts by groups to mobilize stakeholders and the wider public to indirectly influence policy (e.g. Kollman, 1998). This might happen as a reaction to the potential challenges of obtaining insider access. While many interest groups may simultaneously pursue insider and outsider strategies (e.g. Binderkrantz, 2005; Dür & Mateo, 2013) (especially resourceful ones), the choice of how interest groups divide their efforts between insider and outsider channels is likely to be interdependent. Groups that face challenges obtaining access in certain channels can thus be expected to place greater emphasis on expanding efforts in other channels.

As an example, Holyoke (2003) shows that in cases where interest groups cannot match the access of their opponents, they frequently conserve their resources for use in other lobbying channels. Also, Baumgartner and Jones (1993) discuss how the choices of lobbying between different channels are linked. They argue that lobbyists consciously target their efforts in ways that redirect issues from venues where they enjoy less support to those where they enjoy more.

Intensified use of public communications on social media also does not need to be seen as a reaction to changes in access only. Despite the complementarity of these different types of activities, NGOs represent societal interests and are frequently seen as placing relatively more emphasis on outside lobbying than direct communications with policy-makers compared to, for example, business groups (e.g. Gais & Walker Jr, 1991). These more public strategies can be less costly for NGOs, and potentially more effective for them on issues that concern the broader public and have public appeal.

Irrespective of whether social media is used as a means of outsider lobbying, it enables NGOs to reach a larger audience relatively cheaply, to gain the attention of policy-makers, but also to distribute information, to build up communities, and to interact with supporters and members (e.g. Chalmers & Shotton, 2016; Lovejoy & Saxton, 2012, Van der Graaf et al., 2016). Such public campaigns are, for example, also important for NGOs to signal their commitment and to engage with their supporters. According to Kanol and Nat (2017), there is a difference in social media behavior between cause interest groups working to achieve public goals and sectional groups working for the economic benefit of their members. The former, they argue, are more likely to use social media to mobilize the public to act and engage in two-way communication with the public. In their case, NGOs belong to the first category; business interests, to the second. A key reason why social media may be particularly valuable for NGOs to communicate and engage with members and supporters is that these constituencies are typically of a more diffuse and fluent character than is the case for business interests. The threats and uncertainties of the crisis—and the resulting need to minimize physical interaction (e.g. public conferences and meetings)—are likely to have increased their incentive to use social media to reach these types of stakeholders. Moreover, the wide and encompassing scope and high saliency of the crisis may provide relatively strong incentives for NGOs to mobilize their societal constituencies and the wider public via social media. Hence, a pan-societal crisis affecting the vast majority of societal interests may make public communications on social media even more valuable for NGOs than on a standard policy issue affecting more narrow segments of the public.

In this way, NGOs may not only intensify public communications to compensate for difficulties in obtaining insider access to policy-makers. They may also put more emphasis on social media activity in response to a crisis because such activity is deemed particularly valuable to reach their goals and their diffuse group of supporters. Therefore, whereas we expect that business interests increased political access relative to NGOs, we expect the opposite with respect to public communications on social media.

#### Hypothesis 2

The COVID-19 pandemic increased engagement on social media among NGOs relative to business interests.

## Data and Research Design

We test our hypotheses using data from the population of interest groups in the European Union’s official Transparency Register.^6^ Although not a state, the EU adopts decisions that have substantial effects on the daily lives of its 450 million citizens (Hix & Hoyland, 2011). Moreover, while it does not have exclusive competence in policy areas affected by the pandemic, its policy agenda during COVID-19 was dominated by many of the same issues as national political systems, i.e. public health and the socio-economic impact of the crisis.^7^ The roughly 12,000 registered interest groups include actors headquartered not only in EU member states, but also in roughly 90 non-EU countries that have lobbying interests in the EU. The register defines an “interest representative” as “any (natural or legal) person, formal or informal group, association or network that engages in activities [..] with the objective of influencing the formulation or implementation of policy or legislation, or the decision-making processes of EU institutions, bodies, offices and agencies” (the Register does not apply to individual citizens acting in a strictly personal capacity and not in association with others).^8^ In line with the Register, we use a behavioral definition of interest groups referring to “advocates” engaged in observable, policy-related activities as opposed to restricting the term to those with specific organizational characteristics (e.g. membership associations) (Baroni et al., 2014). Interest groups face strong incentives to register in order to participate in consultations and advisory bodies. The Register is updated and has a Secretariat undertaking quality checks of the data.

### Data

Although the COVID-19 pandemic is not representative of all potential crises (Boin et al., 2006), it provides a unique opportunity to clarify the empirical connection between crisis and inequalities in interest representation. Its widespread scope incentivized an exceptionally wide range of actors to mobilize.

To document the pandemic’s effects on differences in political access and communication patterns among business interests and NGOs, we combine data from the Transparency Register with two datasets measuring political access and social media usage. First, we use data compiled by Transparency International of the population of physical and online meetings between interest groups and EU politicians and bureaucrats (Commissioners and high-level Commission civil servants) from January 1, 2019 to September 30, 2020.^9^ These data allow us to create a panel dataset in which each observation indicates the number of meetings a given interest group had with politicians or civil servants in a given month. The panel data thus contain the count of the meetings each group within the Transparency Register had with a policy-maker each month.^10^

In total, interest groups met with EU politicians and bureaucrats 3129 times prior to the pandemic, and 2009 times during the pandemic period under study. The panel data cover the period during which each actor was in the Transparency Register. Our analysis focuses on differences in political access between the two largest categories of interest groups in the register (77% of all groups):

1. “Companies and businesses,” which include companies, consultancies, trade and business associations, and law firms, and
2. “NGOs and identity groups,” which include NGOs, platforms, networks, and organizations representing religious communities (for the coding of business interests and NGOs, see Online Appendix B).

To give a sense qualitatively of the business interests and NGOs in the data that witnessed an increase in meetings with policy-makers in the post-pandemic period, many are related to broad economic interests and the health care sector. For example, among business interests, the Confederation of European Business (BUSINESSEUROPE) and the European Federation of Pharmaceutical Industries and Associations (EFPIA) increased their number of meetings, on average, with policy-makers, and among NGOs, Médecins Sans Frontiéres International (MSF) and the Climate Action Network Europe (CAN Europe) did so. These meetings might concern, for example, “industrial strategy [during the pandemic]” (BUSINESSEUROPE); “shortages of medicines and medical devices” (EFPIA); the “autonomy of the elderly situation in care homes [during the pandemic]” (MSF); and the “priorities for the Coronavirus recovery” (CAN Europe).^11^

Second, we examine the activity of interest groups on social media with Twitter data of all posts since the beginning of 2019 by EU-registered lobbyists with a Twitter account. We first scraped website data from all interest groups before identifying and recording any Twitter account name listed on their official website. We then manually validated each account and conducted a manual search for Twitter accounts missed through web-scraping. Our Twitter account list contains the names of 7846 out of 11,967 registered lobbyists.

Finally, we collected all tweets sent between January 1, 2019 and September 30, 2020. We first collected the most recent 3200 tweets per actor (as limited by Twitter), and then used Twitter’s Premium Search API to fill in the remaining tweets for accounts sending more than 3200 posts in the period. This social media dataset contains 3.6 million tweets from the 14 months prior to the pandemic, and 2 million tweets from the 9 months after the widespread lock-downs began in March, 2020. From these data, we create a panel dataset of the number of tweets sent by each interest group within a given month. In total, the interest groups in the Twitter data span a wide range of businesses and NGOs from 95 of the 116 countries represented in the Transparency Register.

To give examples of the tweets from the business interests and NGOs used as examples above, the subjects related to COVID are similar. For instance, the Confederation of European Business provides information to users about the need for policy-makers to “focus on job creation and growth-enhancing policies” during the COVID recovery; the European Federation of Pharmaceutical Industries and Associations notes its “commitment to work with governments” to find solutions to the pandemic; Médecins Sans Frontiéres International highlights the need to continue “regular health activities safely to ensure those needs are not overlooked”; and Climate Action Network Europe advocates “addressing the [covid-related] economic slowdown by fostering the #EUGreenDeal”.

### Research Design

We estimate the differential effects of the COVID-19 pandemic on NGO and business group access and social media communication using a difference-in-differences strategy. Our baseline difference-in-differences model is specified as follows:

1 $$\begin{aligned} y_{it} = \delta _i + \phi _t + \beta {\text{Pandemic}}_{it} \times \text{NGO}_{it} + \epsilon _{it}, \end{aligned}$$

where $y_{it}$ denotes the outcome variable (the number of meetings or number of tweets) for interest group *i* in month *t*, and $\delta _i$ and $\phi _t$ denote interest group and month fixed effects. Interest group fixed effects allow us to examine within-group variation, and thus account for unobserved differences among groups that do not vary with time. Month fixed effects account for time-varying shocks that affect all groups at once. The variable ${\text{Pandemic}}_{it}$ denotes a binary variable coded 0 for any month before the onset of pandemic lock-downs across the EU (prior to March, 2020), and 1 for any month thereafter.^12^ The variable $\text{NGO}_{it}$ denotes a binary variable coded 1 for NGOs and 0 for business interests.^13^ Our parameter of interest is $\beta$ and captures the pandemic’s differential effect on NGOs relative to business interests. In all models, we cluster standard errors at the interest group level. We note that the model cannot estimate the effect of the pandemic on interest group behavior overall because all units are “treated” after the pandemic’s onset. Instead, the model estimates the difference in interest group behavior between NGOs and business interests as a result of the pandemic.

The model identifies the causal effect of the pandemic under the assumption that trends in the difference between business interests and NGOs for each outcome prior to the pandemic are parallel and would have tracked similarly were it not for the pandemic (Angrist & Pischke, 2009). This counterfactual is fundamentally unknowable. However, we can test for pre-pandemic parallel trends by fitting a model with lags that calculates per-month differences between business interests and NGOs. If the differences in political access and social media posting frequency between NGOs and business interests are effectively equivalent across time, then we have reasonably strong evidence that trends between both sets of interest groups are similar in the pre-pandemic period. We examine this in Online Appendix D. The results suggests that pre-pandemic trends in the frequency of social media posting by NGOs and business interests are parallel, but that NGOs have increasingly made up the difference in access to policy-makers over time. To adjust for this, we use a difference-in-differences model that includes interest-group time trends (Angrist & Pischke, 2009):

2 $$\begin{aligned} y_{it} = \delta _i + \phi _t + \lambda _i \text{t} + \beta {\text{Pandemic}}_{it} \times \text{NGO}_{it} + \epsilon _{it}, \end{aligned}$$

where the additional parameter $\lambda _i$ captures a separate time trend for each interest group *i*.^14^ With this model, differences between business interests and NGOs track similarly across time (see Online Appendix D). As a robustness check, we also fit models in the Results section to the log number of meetings and social media posts, and with count models, which do not substantively change the results (see Appendices I and O). In Online Appendix J, we also fit models with a placebo intervention for exactly a year earlier to the pandemic to rule out that the pandemic occurred at a uniquely beneficial time of year for either business interests or NGOs. As expected, these models present null results.

In addition to our difference-in-differences setup, we also examine the *dynamics* of the effect of the pandemic by fitting an event study model:

3 $$\begin{aligned} y_{it} = \delta _i + \phi _t + \lambda _i \text{t} + {\sum _{t = \text{1}}^{7}}\beta _t \textbf{1}_{t} \times \text{NGO}_{it} + \epsilon _{it}, \end{aligned}$$

where $y_{it}$ denotes the outcome variable for group *i* in month *t*; $\delta _i$ and $\phi _t$ are interest group and month fixed effects; and $\lambda _i$ are interest group-level time trends. Event study models are difference-in-differences models such that rather than a single parameter to measure an effect (as in Eq. 1), the parameters $\beta _t$ capture differences in the outcome variable *per month* after onset of the pandemic ($t \in \{1, 2, \ldots , 7\}$) relative to the time period immediately prior ($t \in \{-13, -12, \ldots , 0\}$). This allows us to investigate the dynamics and duration of each effect.

## Results

**Fig. 1:**
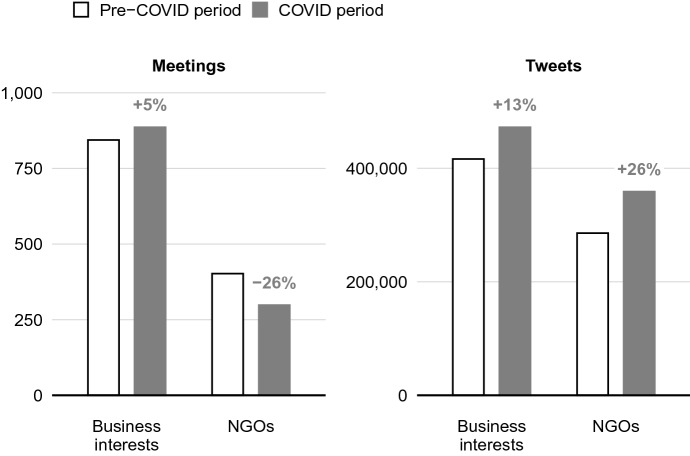
Number of meetings with policy-makers and number of Tweets sent (3 months before and after the pandemic)

We begin by presenting descriptive summaries of the number of meetings that NGOs and business interests had with policy-makers and their number of tweets from 3 months prior to the pandemic, and 3 months afterward. The data in the first panel of Fig. 1 show that business interests saw a 5% increase in the number of meetings with policy-makers when comparing the 3 months immediately prior to the pandemic to the 3 months afterward. NGOs, by contrast, saw a 26% decrease—a large relative difference in changes between businesses ($+$5%) and NGOs (− 26%). In the second panel, we see the reverse in these relative differences: NGOs substantially increased the frequency of their social media posts (by 26%) compared to the relatively smaller increase (13%) among business interests. These descriptive results provide prima facie evidence for our hypotheses, which we now investigate more rigorously.

### The Differential Effects on Meeting Access and Social Media Activity

**Fig. 2:**
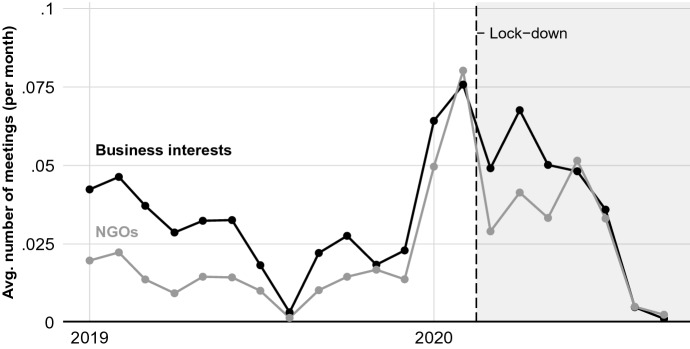
Average number of meetings with EU policy-makers among NGOs and business interests over time

In Fig. 2, we present a comparison of the average number of meetings with policy-makers among NGOs and business interests over time. Prior to the pandemic, there was a decreasing gap in the average number of meetings with policy-makers among NGOs and business interests. Indeed, in the month immediately prior to the pandemic lock-downs, NGOs had more meetings with policy-makers on average than did business groups. One reason is that the number of meetings did not keep apace with the growing number of registered business interest groups over time: more business interests register with the EU per month on average than do NGOs (see Online Appendix A), driving down the average number of meetings.^15^

We estimate the effect of the pandemic on relative access to meetings with policy-makers by fitting a difference-in-differences model as specified in Eq. 2, where the outcome is measured as the number of meetings for an interest group per month.

Consistent with expectations from the first hypothesis, Model (1) of Table 1 indicates that the pandemic caused a decrease in NGOs’ access to meetings with EU policy-makers relative to business interests. On average, we estimate that the pandemic caused a 0.017 decrease in the number of meetings that NGOs had with policy-makers. On its face, the magnitude of this effect may appear small. It is not. As one can see in Fig. 2, the baseline number of meetings any interest group has with policy-makers in a given month is low due to the large number of interest groups.^16^ After the onset of the pandemic, for example, NGOs had, on average, 0.028 meetings with policy-makers per month, compared to 0.035 for business interests^17^ (a 0.008 gap favoring business interests). In other words, business interests received roughly a third more meetings per group on average than did NGOs. Counterfactually, were the effect of the pandemic ($\beta = -0.017$) instead zero, the gap in meetings per interest group would favor NGOs, with NGOs obtaining roughly 0.008 more meetings on average than business interests. The pandemic thus caused a substantial decrease in political representation for NGOs relative to business interests.^18^

**Table 1:** Regression results of the effect of the COVID-19 pandemic on meeting access and social media activity

	Outcome variable	
	Number of meetings	Number of tweets
	(1)	(2)
Lock-down $$\times$$ NGO interest group	− 0.017$$^{***}$$	8.669$$^{**}$$
	(0.005)	(2.873)
Month fixed effect	$\checkmark$	$\checkmark$
Interest group fixed effect	$\checkmark$	$\checkmark$
Interest group time trends	$\checkmark$	$\checkmark$
Observations	163,631	103,886
R$^{2}$	0.295	0.670

*p<0.05; **p<0.01; ***p<0.001. Standard errors in parentheses

**Fig. 3:**
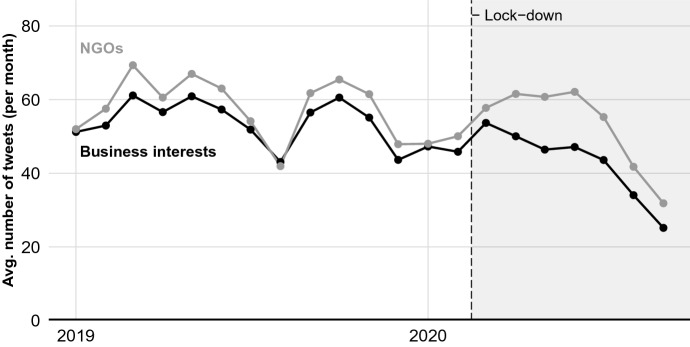
Average number of tweets sent by interest groups representing business interests and NGOs over time

We now investigate the effect of the pandemic on differences between NGOs and business interests in Twitter activity. To begin, we present the average frequency of tweets sent by NGOs and business interests per month from January 2019 to September 2020 in Fig. 3. Unlike the data cataloguing interest groups’ meetings with policy-makers, we observe no clear trends in the differences in the frequency of social media posts between NGOs and business interests. On average, NGOs use social media more frequently than business interests. Given that more business interests are registered with the EU in total, however, the aggregate number of tweets sent by business interests (3.3 million) is substantially larger than that from NGOs (2.3 million).

To estimate the effect of the pandemic on differences in social media posting by NGOs and business interests, we fit the model specified in Eq. 2, where the outcome is the number of Twitter posts sent per interest group per month. Results are presented in Model (2) of Table 1. As the model shows, the pandemic is estimated to have caused an increase in the frequency of social media posts by NGOs relative to business interests. On average, we estimate that the pandemic caused an 8.7 unit increase in the number of posts by NGOs relative to business interests.^19^ To put the magnitude of this effect in context, after onset of the COVID pandemic, the number of tweets sent by business interests was 50.8, and that by NGOs was 60.5 (a 9.7 tweet gap favoring NGOs). NGOs thus sent roughly 19% more tweets on average per interest group than did business interest. Counterfactually, were the effect of the pandemic ($\beta = 8.7$) instead zero, the difference in the average frequency of social media posts per interest would be near zero (1 tweet in favor of NGOs).

### The Dynamic Effects of the Pandemic on the Relative Prominence of Business Interests Versus NGOs

We now investigate the *dynamics* of the pandemic’s effect on political access and social media activity. To do so, we use an event study model (Eq. 3) that allows us to estimate the magnitude of the pandemic’s effect over time by comparing per-month differences between businesses and NGOs relative to figures immediately prior to the pandemic.

**Fig. 4:**
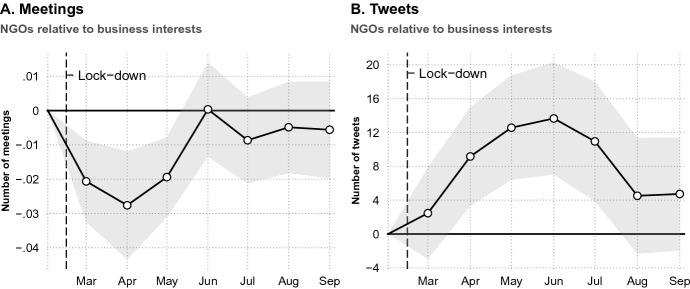
Dynamic effects of the pandemic on meeting access and frequency of social media posts. Shaded area represents 95% CIs. Examination of pre-pandemic differences can be found in Fig. D5 in Online Appendix D

Results from the model with meeting data are visualized in Panel A of Fig. 4 (complete regression table in Online Appendix E). Each point indicates the difference in the number of meetings for NGOs relative to business interests for each month after the onset of the pandemic. In the 3 months after the onset of the pandemic, we observe an immediate drop in the number of meetings for NGOs relative to business interests. These differences rebound after roughly 4 months. Thus, for political access to policy-makers, the advantage of business interests is specific to the early period of—likely highly consequential—policy-making. This finding is not unexpected given our theoretical argument that differences in prominence are driven by decision-makers being able to prioritize output legitimacy over input legitimacy and inclusiveness in stakeholder participation due to the urgency, uncertainty and threat of the pandemic. Hence, as the crisis unfolds, we would expect these factors to play less of a role, stimulating decision-making to return to a mode that resembles ’“normal politics” more with a requirement to ensure broader participation and consultation with stakeholders in the way it was pursued pre-pandemic.

The results from an equivalent model with Twitter data are presented in Panel B of Fig. 4. Here the effect of the pandemic on differences in the frequency of posting to social media by NGOs relative to business interests is roughly analogous, but in the opposite direction: social media activity by NGOs relative to business interests increases in the first months after the onset of the pandemic, but declines sharply thereafter.

**Fig. 5:**
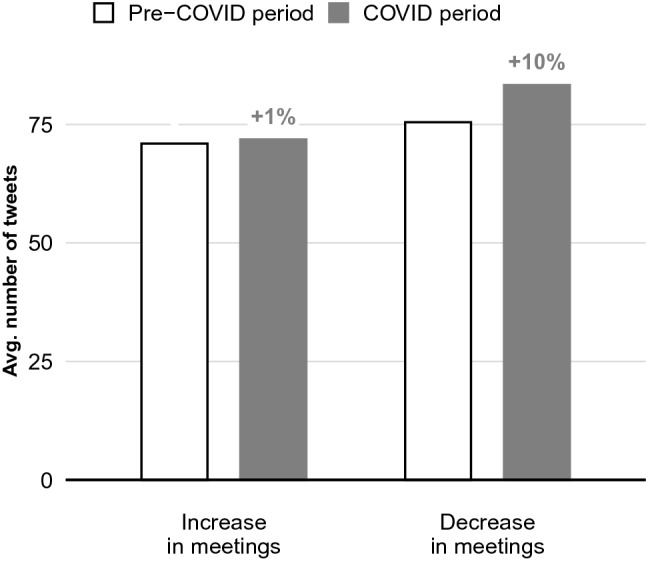
Number of tweets sent immediately before and after the pandemic among interest groups that increased or decreased access to policy-makers

Figure 4 provides suggestive evidence of the interdependence between political access and social media behavior in the months immediately following the onset of the pandemic: that NGOs potentially substituted access to meetings with an increase in public communication. To shed more light on this, we provide a descriptive comparison of the Twitter behavior of interest groups that witnessed a *decrease* in the number of meetings that they had with policy-makers between the pre-COVID and COVID period, and those that witnessed an *increase* in meetings. We do so by calculating the number of tweets sent by each of these groups in the 3 months immediately prior to and 3 month immediately after the onset of the pandemic. Figure 5 presents the results. As the figure shows, those interest groups that saw a decrease in meetings increased the average number of tweets that they sent in the wake of the pandemic by 10%, as compared to an increase of only 1% by those groups that saw an increase in meetings, a difference of 9 percentage points (p $=$ 0.08).

### Evidence from COVID-Specific Meetings and Tweets

To scrutinize the causal impact of the pandemic further, we leverage information about the purpose of each meeting and the contents of each Twitter post. This allows us to investigate whether differences in meeting access and Twitter activity are driven by issues concerning the pandemic itself. We classify political meetings and tweets as being explicitly related to the pandemic by creating a multi-lingual dictionary across 24 languages to code any meeting or social media post concerning the pandemic itself or related terms (e.g. “corona”, “lockdown”, “pandemic”). Applying this dictionary demonstrates that the pandemic resulted in relatively large numbers of meetings related to the issue, with 819 observations during the March-September period of our data (out of a total of 2009 entries). Among tweets, we identify roughly 360,000 tweets from interest groups containing terms related to the pandemic during the relevant period (out of roughly 2 million).

**Table 2:** Regression results of the effect of the pandemic on meeting access and social media activity (COVID-related meetings and tweets removed)

	Outcome variable	
	Number of meetings	Number of tweets
	(1)	(2)
Lock-down $\times$ NGO interest group	− 0.007	4.009
	(0.004)	(2.801)
Month fixed effect	$\checkmark$	$\checkmark$
Interest group fixed effect	$\checkmark$	$\checkmark$
Interest group time trends	$\checkmark$	$\checkmark$
Observations	163,631	103,886
R$^{2}$	0.272	0.665

*p<0.05; **p<0.01; ***p<0.001. Standard errors in parentheses

Because meetings related to the pandemic do not occur in the pre-pandemic period, we cannot directly compare differences in COVID-related meetings before and after the start of the pandemic. Instead, we indirectly document the extent to which the increase in inequality in political access among NGOs relative to business interests is the result of pandemic-specific meetings by excluding them from the dataset and re-fitting the models. This allows us to provide evidence in the spirit of a placebo check to the extent that COVID-related meetings drive the main results: if COVID-specific meetings are the cause of the increased inequality in political access, then their exclusion should result in a smaller (or no) increase in meetings for business interests relative to NGOs in the pandemic period. A natural caveat of our keyword search is that meetings indirectly related to the pandemic might not be classified as such, even if they are partially linked. Nevertheless, observing a smaller or no increase in the gap in political access between NGOs and business interests is suggestive that the mechanism driving the observed effects is due to inequalities concerning access to COVID-related meetings themselves.

We present the results of a difference-in-differences model in Table 2 where the outcome variable is the number of meetings with EU policy-makers, and all meetings are included except those classified as concerning the pandemic in Model (1) of Table 2. We find no strong evidence that the pandemic widened the gap in political access between NGOs and business interests when meetings specifically concerning the pandemic are removed from the data. The effect size is less than half compared to what was observed in Model (1) of Table 1 in which COVID-19 Tweets were included.

We conduct a similar analysis for differences in social media activity between NGOs and business interests. As with the meetings data, we exclude all tweets related to the pandemic and re-fit the model. Results are presented in Model (2) of Table 2. Analogous to the access results, we find no strong evidence that the pandemic differentially affected the frequency of social media posts between NGOs and business interests when COVID-related posts are excluded.

Overall, the results suggest that the decrease in access to political meetings among NGOs relative to business interests was the result of inequalities in access to meetings concerning policies linked to the pandemic itself. Similarly, social media results suggest that the relative increase in the frequency of posts by NGOs was driven by an increased social prominence of these groups in pandemic-related content.

### Additional Analyses

We investigate whether the differential effect of the pandemic on NGOs and business interests is consistent across the policy areas in which the interest groups in the Transparency Register declare to work (e.g. those concerned with public health, business & industry, environmental regulation, etc.). Sub-group analysis necessarily reduces statistical power, but finding consistency in the estimated effects across sub-fields would provide additional evidence of a broad effect in favor of business groups. As we show in Online Appendix L, this is indeed the case. We demonstrate that the pandemic differentially benefited business interests at the expense of NGOs in meetings across a variety of topic portfolios of the interest groups. Large differences are found among economic and public health businesses, although the reduction in statistical power prevents precise comparisons in the effects across sub-fields. Conversely, we find consistent effects that the pandemic differentially increased the social media posting frequency of NGOs relative to business interests. In Online Appendix R, we also show that the effect of the pandemic on NGOs’ access to meetings is negative relative to both companies and other business interest groups, and its effect on NGOs’ tweets is positive relative to companies and other business interest groups (but only significantly so for the former).

Finally, we address whether differences in political access and social media activity between NGOs and business interests are driven by existing differences in resource availability: that business interests’ privileged access to resources gave them the upper hand in accessing policy-makers during the crisis. To measure resources, we use data on lobbying resources for each registered interest group from the EU’s Transparency Register. We classify each interest group as having a “high” level of resources if they are in the upper tercile of lobbying expenditures, as compared to interest groups in the bottom terciles (“low”). Because the upper tercile as a cutoff is arbitrary, we run a set of robustness checks for the results below using a wide array of cutoff values (see Appendices F and G); make comparisons between only the most resource-rich (upper quartile) and least-resource rich (lower quartile) (Online Appendix F and G); and examine interest group staff size as an alternative resource measure (Online Appendix H). Results are not substantively different across any of these checks.

We begin by investigating whether the finding that business interests gained preferential access to policy-makers at the expense of NGOs differed among high- and low-resource groups. To do this, we first estimate the effect of the pandemic on political access to meetings among high-resource NGOs relative to high-resource business interests. Results are presented in Model (1) in Table 3. Consistent with our main findings, for interest groups with large lobbying budgets, we still observe that the pandemic caused a decrease in (high-resource) NGOs’ access to meetings with policy-makers relative to business interests. We then fit the model to meetings data from interest groups with low lobbying resource budgets. The results, presented in Model (2) of Table 3, show no strong evidence (p $=$ 0.24) that the pandemic caused such a decrease among low-resource interest groups. In sum, when the data are stratified by resources, we see that it is largely well-resourced businesses that benefited from political access as a result of the pandemic at the expense of well-resourced NGOs.

**Table 3:** Regression results of the effect of the COVID-19 pandemic on meeting access and social media activity, stratified by interest group resource levels

	Outcome variable			
	Number of meetings		Number of tweets	
	(1)	(2)	(3)	(4)
Lock-down $$\times$$ NGO interest group	− 0.044$$^{***}$$	− 0.003	7.421$$^{**}$$	10.199$$^{*}$$
	(0.013)	(0.003)	(2.529)	(5.071)
Month fixed effect	$\checkmark$	$\checkmark$	$\checkmark$	$\checkmark$
Interest group fixed effect	$\checkmark$	$\checkmark$	$\checkmark$	$\checkmark$
Interest group time trends	$\checkmark$	$\checkmark$	$\checkmark$	$\checkmark$
Data	High resource	Low resource	High resource	Low resource
	Groups	Groups	Groups	Groups
Observations	58,390	102,963	44,773	57,955
R$^{2}$	0.308	0.151	0.675	0.665

*p<0.05; **p<0.01; ***p<0.001. Standard errors in parentheses

Similarly, we examine data from the social media posts of NGOs and business interests similarly by stratifying by resource levels in Models (3) and (4) in Table 3. Consistent with our main findings, NGOs with both high and low levels of resources increased the frequency of their communications on social media relative to that of high-resource and low-resource business interests. In sum, the hypothesized difference between NGOs and business interests generally do not change when stratifying by resource level.

We supplement these analyses by testing whether the pandemic increased political access among interest groups with higher resources relative to those with lower resources, regardless of whether they are NGOs or business interests. The results (see Online Appendix F) do not indicate that this is the case.

In sum, we find that the pandemic’s effect on differences between business interests and NGOs in Twitter activity and meeting access are observed broadly across sub-fields and unlikely to be driven by differences in the resources available to these types of interest groups *in general*. This suggests that inequality in access that favored business groups was not the result of pre-existing disparities that allowed business interests to respond more quickly than NGOs in their efforts to gain access.

## Conclusion

Inequalities in interest representation have long worried political observers and academics, and have frequently been viewed as a persistent feature of policy-making, both across political systems and across time (Rasmussen & Reher, Forthcoming). The COVID-19 pandemic provides a unique opportunity to shed light on whether large-scale shocks to the political agenda can magnify or reduce these inequalities due to its pan-societal nature and its effects on a broad set of different interests (Furnas et al., 2021).

Our results demonstrate that the COVID-19 crisis caused substantial increases in direct access to policy-makers among business interests vis-a-vis NGOs, a finding that is consistent with interest group’s own perceptions of the effects of the pandemic (Junk et al., 2021). By contrast, the crisis caused large increases in the social media prominence of NGOs relative to business interests. Furthermore, with information about the subject of meetings and the text of tweets, we show that these changes during the first crucial months of the pandemic were likely driven by changes in political access and communications specific to the crisis.

From a normative point of view, these results offer both good and bad news. On the one hand, what looks like a strengthening of business prominence in insider lobbying in access to policy-makers was primarily an issue in the early phase of the pandemic and is not replicated in public communication on social media. The fact that our findings are unlikely to be driven by differences in economic resources between NGOs and business interests can also be seen as positive news. Among NGOs and business interests with similarly large resource endowments, we find that business interests still gain substantial access to policy-makers relative to NGOs. Moreover, for social media activity, NGOs increase their activities relative to business interests both among low-resource and high-resource interest groups. This finding complements recent work by Furnas et al. (2021) showing that money is not a key factor determining attention by Members of Congress to interest group concerns during COVID-19. They find only a weak association between campaign donations of a sector and subsequent mentioning of the sector in press releases by these members (Furnas et al., 2021).

On the other hand, our results also raise concerns. First, the two channels of activity examined are unlikely to equally affect the political decisions on, for instance, emergency legislation, rescue packages, or regulations regarding the reopening of society. Direct access to policy-makers, on its face, gives some organized interests privileged access over others. While access to policy-makers is no guarantee for actual influence, such access is probably a more straightforward way to influence decisions than through increased public communication on, for example, social media. As emphasized, social media activity may also not only be directed at lobbying, but also at maintaining relations to constituents etc. At the same time, we need to be careful drawing too strong normative conclusions based on our findings. While we do not have evidence to suggest that either business interests or NGOs as a whole are more affected by the crisis, we cannot rule out that the urgency of the problems dealt with during the pandemic pushed policy-makers to be more one-sided in the advice sought when adopting specific decisions.

By broadly affecting the interests of an exceptionally wide range of actors, the COVID-19 crisis provided us a unique opportunity to clarify the empirical connection between crisis and inequality in interest representation between business interests and NGOs since both types of interests were strongly affected by the crisis. Yet, what makes the crisis a clarifying event in terms of inequality in interest representation also leads to questions whether our arguments apply in smaller-scale, less wide-ranging, or different types of crises. This is an important avenue for future empirical research. We believe that the tendency of decision-makers to prioritize output legitimacy over input legitimacy and inclusivity in decision-making will be present in decision-making during crises more broadly. Nevertheless, the character of a specific crisis will likely affect which interests policy-makers prioritize, i.e. whether it is an economic crisis, security crisis, etc. In addition, more intense or wide-ranging crises like the COVID-19 pandemic might, for example, be expected to provide more political flexibility to policy-makers to whom they provide access. It may also place higher pressure on policy-makers to privilege interest groups that can help them with information needed to adopt fast decisions to alleviate the severity of the crisis.

Furthermore, although the EU shares similarities with other political systems and has discussed many of the same issues as national governments in its response to COVID-19, there is room for further comparative research to test the external validity of our findings regarding insider access. It would also be useful to conduct further content analysis of the tweets and obtain more information about effects of the pandemic, not only on who *obtained* but also who *requested* access to draw more fine-grained normative implications of the identified patterns. Relatedly, it will be important to scrutinize whether changes in access during the pandemic ultimately affect policy itself. What is clear, however, is that a large-scale crisis such as the COVID-19 pandemic can have substantial effects on how strongly different types of interests are positioned vis-a-vis both policy-makers and the public.

## Supplementary Information

Below is the link to the electronic supplementary material.

Supplementary file1 (PDF 471 kb)

## Data Availability

Replication material is available at https://doi.org/10.7910/DVN/BO3GVG.

## Footnotes

1 https://www.nytimes.com/2020/03/28/us/politics/coronavirus-money-lobbyists.html.

2 https://www.consilium.europa.eu/en/policies/eu-recovery-plan/.

3 See e.g. https://corporateeurope.org/en/2020/05/corona-lobby-watch.

4 See e.g. https://carbonmarketwatch.org/wp-content/uploads/2020/05/Never-wasting-a-crisis-1.pdf.

5 These two actor categories constitute the vast share of actors (77%) in our dataset (detailed below). At the same time we also conduct a series of robustness checks where we include other group types, i.e. trade unions and organizations representing subnational authorities in our comparisons (see Online Appendix M).

6 List collected in October, 2020.

7 https://ec.europa.eu/info/live-work-travel-eu/coronavirus-response_en.

8 https://ec.europa.eu/transparencyregister/public/staticPage/displayStaticPage.do?locale=en &reference=GUIDELINES.

9 Even if the dataset does not account for all possible lobbying activities with regard to EU policy, it is by far the largest available dataset of meetings between the EU and external interests and the European Commission played a key role during the pandemic, coordinating the EU’s COVID-19 response. Moreover, lists of meetings with lower levels of the European Commission, and staff from other EU institution and national administrations are not publicly available

10 If a group within the Transparency Register population had no meetings through the time period of interest, they are coded as having 0 meetings in each month. Interest group fixed effects account for this fact, as described in our model specification.

11 In Online Appendix Q, we provide a list of the interest groups that saw the largest increases in the average number of meetings with policy-makers.

12 Strict regulations concerning the crisis began in March, 2020 (see Online Appendix C).

13 Separate component terms for the variable in the interaction ${\text{Pandemic}}_{it} \times \text{NGO}_{it}$ are excluded because they are perfectly correlated with the unit and time fixed effects.

14 Results are effectively equivalent with group-type level trends (business/NGO) rather than unit-level trends.

15 Online Appendix D shows that inclusion of interest group time trends accounts for any absence of parallel trends resulting from this.

16 The low numbers of meetings in August 2019 and 2020 is due to the European summer vacation period.

17 For an examination of differences among business interests by sector, see Online Appendix P.

18 In Online Appendix K, we fit the model to data only from interest groups with a Twitter account to allow for a direct comparison with the social media results below, with effectively equivalent results.

19 In Online Appendix N, we examine the effects of the pandemic on whether tweets are shared and “favorited” more frequently, with similar results.

## References

[CR1] Angrist JD, Pischke J-S (2009). Mostly harmless econometrics: An empiricist’s companion.

[CR2] Baroni L, Carroll BJ, Chalmers AW, Marquez LMM, Rasmussen A (2014). Defining and Classifying Interest Groups. Interest Groups and Advocacy.

[CR3] Baumgartner FR, Jones BD (1993). Institutions and instability in American politics.

[CR4] Baumgartner FR, Berry JM, Hojnacki M, Kimball DC, Leech B (2009). Lobbying and policy change: Who wins, who loses, and why.

[CR5] Bekker M, Ivankovic D, Biermann O (2020). Early lessons from COVID-19 response and shifts in authority: Public trust, policy legitimacy and political inclusion. The European Journal of Public Health.

[CR6] Bentley AF (1908). The process of Government: A study of social pressures.

[CR7] Birkland TA (1998). Focusing events, mobilization, and agenda setting. Journal of Public Policy.

[CR40] Binderkrantz A (2005). Interest Group Strategies: Navigating between Privileged Access and Strategies of Pressure. Political Studies.

[CR8] Boin A, Hart P, Stern E, Sundelius B (2006). The politics of crisis management. Public leadership under pressure.

[CR9] Bouwen P (2004). Exchanging access goods for access: A comparative study of business lobbying in the European Union Institutions. European Journal of Political Research.

[CR10] Broscheid A, Coen D (2007). Lobbying activity and Fora creation in the EU: Empirically exploring the nature of the policy good. Journal of European Public Policy.

[CR41] Chalmers AW, Shotton PA (2016). Changing the face of advocacy? Explaining interest organizations’ use of social media strategies. Political Communication.

[CR11] Coen D (2007). Empirical and theoretical studies in EU lobbying. Journal of European Public Policy.

[CR39] Crepaz, M., Junk, W. M., Hanegraaff, M., & Berkhout, J. (2022). *Viral Lobbying: Strategies, Access and Influence during the COVID-19 Pandemic*. De Gruyter.

[CR12] De Bruycker I (2016). Pressure and expertise: Explaining the information supply of interest groups in EU Legislative Lobbying. JCMS: Journal of Common Market Studies.

[CR42] De Bruycker I, Rasmussen A (2021). Blessing or curse for congruence? How interest mobilization affects congruence between citizens and elected representatives. Journal of Common Market Studies.

[CR36] der Graaf V, Amber SO, Rasmussen A (2016). Weapon of the Weak? The social media landscape of interest groups. European Journal of Communication.

[CR13] Dür A, Mateo G (2013). Gaining access or going public? Interest group strategies in five European Countries. European Journal of Political Research.

[CR14] Eising R (2007). Institutional context, organizational resources and strategic choices. European Union Politics.

[CR43] Flöthe L, Rasmussen A (2019). Public voices in the heavenly chorus? Group type bias and opinion representation. Journal of European Public Policy.

[CR15] Furnas AC, Crosson JM, Lorenz GM (2021). Pandemic pluralism: Legislator championing of organized interests in response to COVID-19. Journal of Political Institutions and Political Economy.

[CR16] Gais TL, Walker Jr JL (1991). Pathways to influence in American politics.

[CR17] Gilens M, Page BI (2014). Testing theories of American politics: Elites, interest groups, and average citizens. Perspectives on Politics.

[CR18] Gray V, Lowery D (2000). The population ecology of interest representation.

[CR19] Hix S, Hoyland B (2011). The political system of the European Union.

[CR20] Holyoke TT (2003). Choosing battlegrounds: Interest group lobbying across multiple venues. Political Research Quarterly.

[CR46] Junk WM (2019). Representation beyond people: Lobbying access of umbrella associations to legislatures and the media. Governance.

[CR21] Junk WM, Crepaz M, Hanegraaff M, Berkhout J, Aizenberg E (2021). Changes in interest group access in times of crisis: No pain, no (lobby) gain. Journal of European Public Policy.

[CR22] Kanol D, Nat M (2017). Interest groups and social media: An examination of cause and sectional groups’ social media strategies in the EU. Journal of Public Affairs.

[CR23] Kollman K (1998). Outside lobbying. Public opinion and interest group strategies.

[CR24] Lovejoy K, Saxton GD (2012). Information, community, and action: How nonprofit organizations use social media. Journal of Computer-Mediated Communication archive.

[CR25] Lowery D, Baumgartner FR, Berkhout J, Berry JM, Halpin D, Hojnacki M, Klüver H, Kohler-Koch B, Richardson J, Schlozman KL (2015). Images of an unbiased interest system. Journal of European Public Policy.

[CR26] Mahoney C (2004). The Power of Institutions. State and Interest Group Activity in the European Union. European Union Politics.

[CR27] Mahoney C, Beckstrand MJ (2011). Following the money: European Union Funding of Civil Society Organizations. JCMS: Journal of Common Market Studies.

[CR44] Rasmussen A, Binderkrantz AS, Klüver H (2021). Organised interests in the media and policy congruence: The contingent impact of the status quo. European Journal of Political Research.

[CR29] Rasmussen A, Carroll B (2014). Determinants of upper-class dominance in the heavenly chorus: Lessons from European Commission Online Consultations. British Journal of Political Science.

[CR30] Rasmussen A, Gross V (2015). Biased access? Exploring selection to advisory committees. European Political Science Review.

[CR45] Rasmussen, A., & Reher, S. (2022). (Inequality in) interest group involvement and the legitimacy of policy-making. *British Journal of Political Science*, OnlineFirst.

[CR31] Scharpf FW (2009). Legitimacy in the multilevel European polity. European Political Science Review.

[CR32] Schattschneider EE (1960). The Semisovereign People.

[CR33] Schlozman KL, Verba S, Brady HE (2012). The unheavenly chorus: Unequal political voice and the broken promise of American democracy.

[CR34] Strickland JM (2020). The declining value of revolving-door lobbyists: Evidence from the American States. American Journal of Political Science.

[CR35] Truman DB (1951). The Governmental Process.

[CR37] Winfield, M. (2020). Governments Must Resist Coronavirus Lobbying and Focus on Long-term Transformation. In *The Conversation*. Retrieved May 14, from https://theconversation.com/governments-must-resist-coronavirus-lobbying-and-focus-on-long-term-transformation-138178

[CR38] Witko C (2006). PACs, issue context, and congressional decisionmaking. Political Research Quarterly.

