# Supplementary material for: The Unequal Effects of the COVID-19 Pandemic on Political Interest Representation
Source: Polit Behav. 2022 Dec 31:1–25. Online ahead of print. doi: 10.1007/s11109-022-09842-x (PMC9803882; doi:10.1007/s11109-022-09842-x)
Supplement: Supplementary file 1 — Supplementary file1 (PDF 471 kb) [file 11109_2022_9842_MOESM1_ESM.pdf]

Online Appendix for

"The Unequal Effects of the COVID-19 Pandemic on Political Interest Representation"

Gregory Eady and Anne Rasmussen

Contents

A The composition of registered interest groups over time 3

B Interest group type definitions and examples 5

C Timing of the pandemic 6

D Examination of pre-treatment parallel trends 7

E Regression results from event study model 11

F Differential effect of the pandemic on high- and low-resource interest groups 13

G Sensitivity of results stratified by resources (Table 3) to alternative codings of 'high' and 'low' resources 18

H Interest group staff size as an alternative measure of resources 21

I Regression results for the log number of meetings and tweets 23

J Placebo intervention 26

K Meetings results among groups with Twitter accounts 27

L Differential effects of the pandemic by sub-category 28

M Differential effects on trade unions and public authorities 32

N Effects on the frequency of sharing social media posts from NGOs and business interests 35

O Poisson regression results 37

P Differences in meetings before and during the COVID pandemic between business interest sectors 40

Q Interest groups that saw largest increase in meetings during the pandemic 43

R Differential effects of the pandemic by companies and other business interest groups 43

A The composition of registered interest groups over time

As noted in the main article, the number of business interests registered in the EU Transparency Register is larger than that of NGOs. Furthermore, in general, there are more business interests added each month to the registry than there are NGOs. To show this, we present in Figure A1 the number of interest groups from each group type registered with the EU over time. As the figure shows, the growth in the number of registered companies and businesses is outpacing that of its NGO counterpart. Growth in the number of business interests and NGOs is roughly proportional to size, as suggested by the parallel lines when these data are presented on the log scale in Figure A2. These differences in growth likely partly explain why there is a decreasing gap over time in the *average* number of meetings that business interests have with EU policy-makers over time relative to NGOs in Figure 2 in the article. The number of meetings that policy-makers have with business interests, in other words, has not kept pace with the growth in the number that register as lobbyists with the EU.

Figure A1: Change in the composition of registered interest groups over time

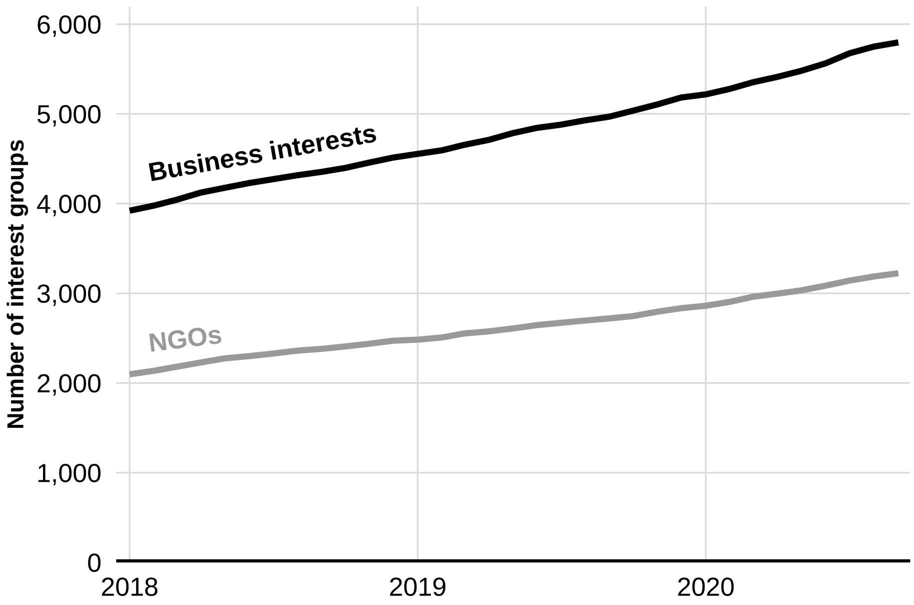

This figure shows the number of business and NGO interest groups that are registered with the EU over time.

Figure A2: Change in the composition of registered interest groups over time (log scale)

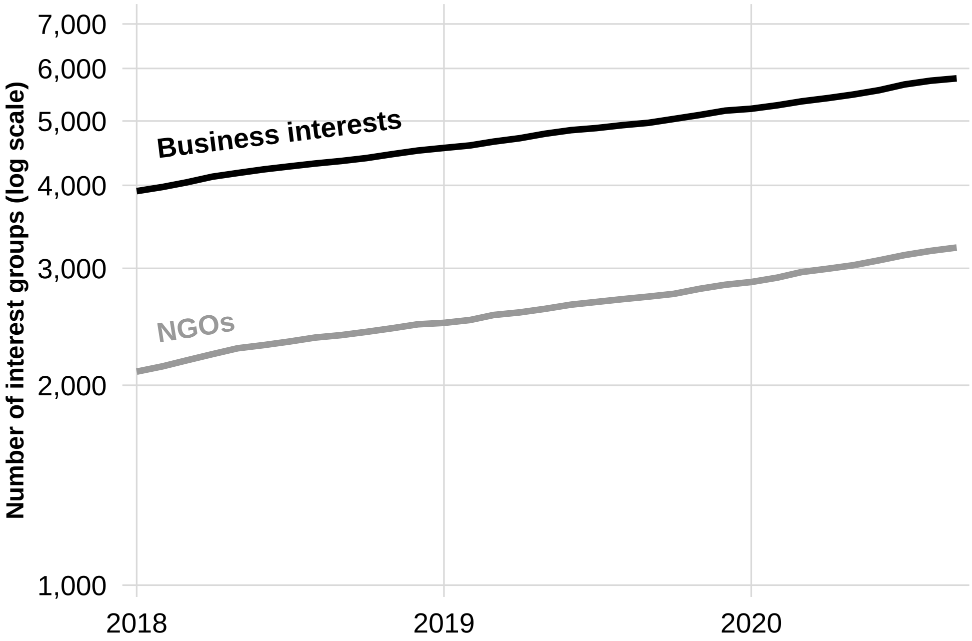

This figure shows the number of business and NGO interest groups that are registered with the EU over time, as graphed on the log scale on the horizontal axis.

B Interest group type definitions and examples

In the main article, we examine interest groups defined as "Business interests" and "NGOs". In the Transparency Register, each interest group is classified internally as belonging to one of fifteen sub-groups. These sub-groups classifications are themselves selected by each interest group when they register as a lobbyist with the EU. The classification of each interest group, therefore, is defined by the group itself, although subject to checks by the Registry secretariat. To examine differences in business interests and NGOs, we therefore collapse the relevant smaller categories into larger ones that define "Business interests" and "NGOs". Our definition, based on these sub-categories, is presented in Table B1.[1]

Table B1: Definition of interest group types

**Category**	**Sub-categories**
Companies & business associations	Companies & groups<br>Professional consultancies<br>Self-employed consultants<br>Law firms<br>Trade and business associations
NGOs & identity groups	Non-governmental organisations, platforms and networks and similar<br>Organisations representing churches and religious communities

Interest group sub-categories not included in these larger groups are "Academic institutions", "Other public or mixed entities created by law whose purpose is to act in the public interest", 'Trade unions and professional associations", "Other sub-national public authorities", "Regional structures", "Think tanks and research institutions", "Transnational associations and networks of public regional or other sub-national authorities", and "Other organisations".

[1]Professional consultancies and self-employed consultants are included among business interests. They may occasionally work for NGOs, however they also form only a small percentage (6%) of interest groups overall.

C Timing of the pandemic

In the article, we code March, 2020 as the beginning of the pandemic. We do so because, first, March is the month in which the World Health Organization declared the pandemic as such (March 11) and, second, March clearly marks the start of widespread governmental responses to the pandemic across the EU, with restrictions on social and economic activities. To show the latter empirically, we use data from the Oxford University Blavatnik School of Government's "Coronavirus Government Response Tracker" (Blavatnik School of Government, 2021). We aggregate the Tracker's "Stringency Index"—a measure of the intensity of government regulations to combat the pandemic—at the level of the EU, and present this measure graphically in Figure C3. As the figure makes clear, widespread governmental responses within the EU ramped up heavily in March, close to the WHO's declaration of the crisis as a pandemic.

Figure C3: COVID-19 Stringency Index across time in the EU

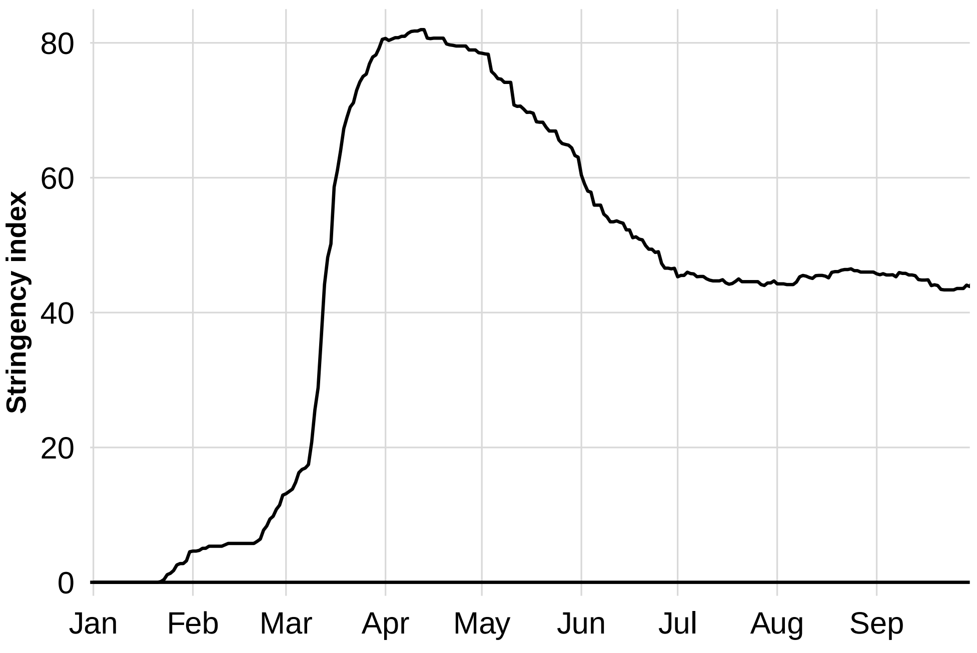

This figure presents a measure of the intensiveness of EU member states' COVID-19 regulations ("stringency index") over time from the Oxford University Blavatnik School of Government's "Coronavirus Government Response Tracker." (Blavatnik School of Government, 2021). Data presented are the average stringency index across all EU member states.

D Examination of pre-treatment parallel trends

Difference-in-differences models rely on an assumption of parallel trends: that prior to an intervention, the outcome variable for the groups of interest move in sync and that, counterfactually, these trends would continue in parallel were it not for the intervention of interest. This counterfactual is, by definition, unknowable. However, it useful to examine whether there are parallel trends in the pre-intervention period: doing so does not provide direct evidence that trends in outcomes would have evolved similarly between groups in the absence of an intervention, but it provides indirect evidence that this assumption is likely reasonable (Cunningham, 2021).

To examine this empirically, we fit difference-in-differences models that include time period leads, such that we calculate separate difference-in-difference estimates for each month *prior* to the pandemic (Angrist and Pischke, 2009; Cunningham, 2021). If the assumption of parallel trends holds, we should observe no systematic difference in the differences between NGOs and business interests month-over-month prior the pandemic.

We include leads in a baseline difference-in-differences model, and one that is more flexible with respective to time trends through the inclusion of additional interest group-level time trends. More formally, our estimating equations are the following:

$$y_{it} = \delta_i + \phi_t + \sum_{t=-13}^{0} \beta_t \text{NGO}_{it} + \epsilon_{it} \tag{D1}$$

$$y_{it} = \delta_i + \phi_t + \lambda_i \text{t} + \sum_{t=-13}^{0} \beta_t \text{NGO}_{it} + \epsilon_{it} \tag{D2}$$

where y_{it} is the outcome variable for group i in month t; δ_i and ϕ_t are interest group and month fixed effects; and λ_i (Equation D2) are interest group-level time trends. In these models, our parameters of interest are β_t, which capture the differences in differences between NGOs and business interests *per month* prior to the pandemic. Estimating separate β_t per

month prior to the pandemic allows us to compare whether the per-month differences between NGOs and business interests differ from each other relative to a baseline month, chosen here as the month immediately prior to the pandemic. If trends between NGOs and business interests are parallel, we should observe no meaningful differences across the range of the estimates of β_t. As noted above and as shown in Equation D1 and Equation D2, we fit these models both with and without group-level time trends, the latter of which flexibly accounts for trends among each interest group in the number of meetings or social media posts over time.

Results from the model for the number of meetings with policy-makers are presented in Figure D4. As Panel A shows, there is evidence that, in the pre-pandemic period, NGOs had less access to meetings with policy-makers (relative to business interests) as compared to later months. Estimates from the first months of the data, for example, show significant differences in access to policy-makers of NGOs relative to business interests that were larger relative to the baseline month immediately prior to the onset of the pandemic. This is also observable descriptively in Figure 1 from the main article, in which the gap between the average number of meetings between NGOs and business interests is decreasing over time. In other words, there appear to be deviations from parallel trends. We can adjust for this, however, by including interest group-specific time trends (Angrist and Pischke, 2009; Cunningham, 2021), as in the model specified in Equation D2. Accordingly, Panel B of Figure D4 presents results for pre-pandemic difference-in-differences from the model with interest group-specific time trends. As Panel B shows, the inclusion of these time trends results in pre-pandemic differences that show no clear changes month-over-month. As we note in the main article, we therefore use as our model for the political meetings data one that includes interest group-specific time trends to flexibly adjust for these differences over time.

We conducted similar tests for the model fit to the data on the number of tweets sent by NGOs and business interests in the pre-pandemic period. Results from these models are

Figure D4: Parallel trends test for differences in the average number of meetings with policy-makers among NGOs and business interests prior to pandemic

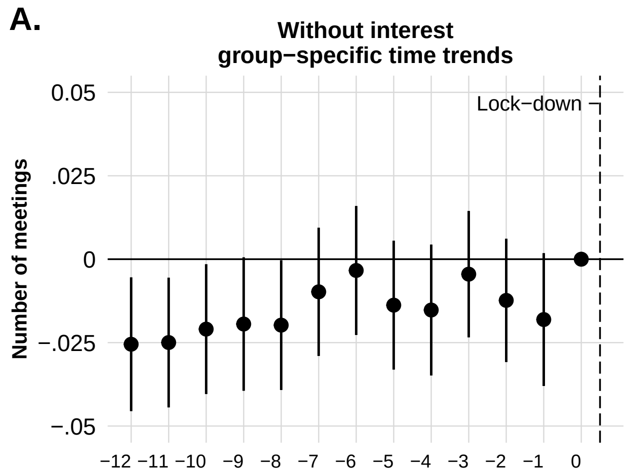

This figure presents estimates of per-month differences between the number of meeting with policy-makers among NGOs relative to business interests prior to the pandemic, where the baseline for comparison is $t = 0$ (i.e. February, 2020)

Figure D5: Parallel trends test for differences in the average number of tweets sent by NGOs and business interests prior to pandemic

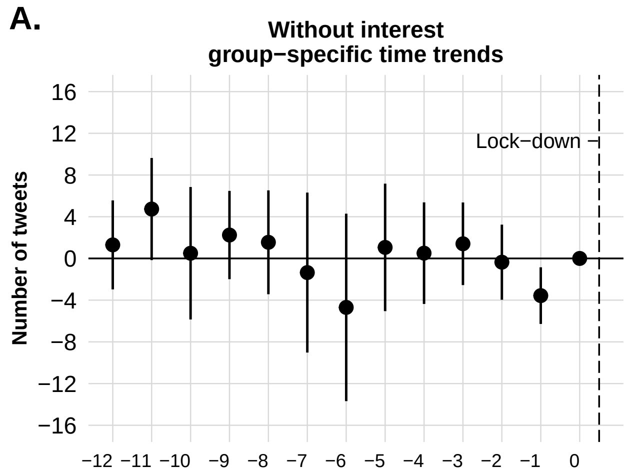

This figure presents estimates of per-month differences between the number of tweets sent by NGOs relative to business interests prior to the pandemic, where the baseline for comparison is $t = 0$ (i.e. February, 2020).

presented in Figure D5. Unlike with the political meetings data, in Panel A of Figure D5, we see no systematic differences in trends that suggest an absence of parallel trends. In Panel B, which presents estimates for a model with interest group-specific time trends, we

also observe no clear pattern. Indeed, estimates in both panels are extremely similar. The more flexible model that includes interest group-level trends, in other words, is performing minimal adjustment. In the main article we include interest group-level time trends when investigating the effect of the pandemic on differences in posting behavior about NGOs and business interests. However, as is consistent with the results in both panels of Figure D5, the results are effectively equivalent in models that do no include interest group-level time trends (not shown).

E Regression results from event study model

In Figure 4 in the main article, we show graphically the results of an event study model specified as follows:

$$y_{it} = \delta_i + \phi_t + \lambda_i \text{t} + \sum_{t=1}^{7} \beta_t \mathbf{1}_t \times \text{NGO}_{it} + \epsilon_{it}, \tag{E3}$$

where y_{it} denotes the outcome variable for group i in month t; δ_i and ϕ_t are interest group and month fixed effects; and λ_i are interest group-level time trends. As we note in the Research Design section of the article, the set of parameters, β_t, capture differences in the outcome variable per month after onset of the pandemic ($t \in \{1, 2, \ldots, 7\}$) relative to the time period prior to the pandemic ($t \in \{-13, -12, \ldots, 0\}$). In Table E2, we present the relevant regression table, where each parameter represents the difference-in-differences for NGOs relative to business interests in a given month. As shown in Figure 4 in the main article, these parameters demonstrate the dynamics of the effect over time.

Table E2: Event study regression results

	DV	
	ln Number of meetings	ln Number of tweets
	(1)	(2)
March, 2020 × NGO interest group	−0.021***	2.498
	(0.006)	(2.797)
April, 2020 × NGO interest group	−0.028***	9.291**
	(0.008)	(3.009)
May, 2020 × NGO interest group	−0.019**	12.779***
	(0.006)	(3.180)
June, 2020 × NGO interest group	0.0004	13.954***
	(0.007)	(3.415)
July, 2020 × NGO interest group	−0.009	11.329**
	(0.006)	(3.645)
August, 2020 × NGO interest group	−0.005	4.982
	(0.007)	(3.522)
September, 2020 × NGO interest group	−0.006	5.281
	(0.007)	(3.440)
Observations	164,541	103,060
R^2	0.295	0.670

*p<0.05; **p<0.01; ***p<0.001. Standard errors, in parentheses, are clustered at the level of the interest group.

F Differential effect of the pandemic on high- and low-resource interest groups

In the main article, we discuss findings concerning the pandemic's differential effects on access to policy-makers and social media among interest groups with 'high' resources (upper tercile) and 'low' resources (lower terciles). We present the complete regression tables in Table F3. As shown in Model (1), we find no evidence of a differential effect of resources on interest groups' access to policy-makers in general ($p = 0.48$). In other words, when pooling data from NGOs and business interests, we find no difference in access to policy-makers among interest groups with low and high levels of resources in general.

In Models (2) and (3), we examine the role of resources *within* interest group types (NGOs and business interests). In Model (2), we find that the pandemic caused an increase in access to policy-makers among business interests with higher resources relative to business interests with lower resources. In Model (3), we find that among NGOs, the pandemic caused a decrease in access to policy-makers among high-resource interest groups relative to low-resource interest groups. This result can be viewed in light of the fact that low-resource NGOs obtain very few meetings with policy-makers to begin with. High-resource NGOs, in other words, became more similar to low-resource NGOs; high-resource business interests, by contrast, gained even greater access relative to their low-resource counterparts.

Finally, we examine the overall role of resources on social media activity in Models 4-6. We find no evidence that the pandemic differentially caused differences in the frequency of posting among business interests and NGOs collectively (Model (1)), or whether comparing high-resource and low-resource interest groups among business interests (Model (2)) and NGOs separately (Model (3)).

Table F3: Regression results of the differential effect of the pandemic on high-resource and low-resource interest groups

	Outcome variable					
	Number of meetings			Number of tweets		
	(1)	(2)	(3)	(4)	(5)	(6)
Lock-down × Resources	0.005	0.017*	−0.024*	−1.268	−1.223	1.153
	(0.007)	(0.009)	(0.010)	(2.066)	(2.483)	(2.987)
Month fixed effect	✓	✓	✓	✓	✓	✓
Interest group fixed effect	✓	✓	✓	✓	✓	✓
Interest group time trends	✓	✓	✓	✓	✓	✓
Data	Businesses & NGOs	Businesses	NGOs	Businesses & NGOs	Businesses	NGOs
Observations	100,309	64,450	35,859	66,618	41,424	25,194
R^2	0.308	0.307	0.309	0.703	0.671	0.779

*p<0.05; **p<0.01; ***p<0.001. Standard errors, in parentheses, are clustered at the level of the interest group. The outcome variable is defined as the number of meetings or tweets from each interest group aggregated at the month level from January 1, 2019 to September 30, 2020.

F.1 Sensitivity to alternative codings of 'high' and 'low' resources

As noted in the article and above, we define interest groups with 'high' resources as those in the upper tercile (above the 66.6th percentile) of all business interests and NGOs, and those with 'low' resources, those interest groups in the bottom two terciles. This choice, however, is nevertheless relatively arbitrary. To test the extent to which the results concerning resources above are sensitive to the coding of groups with 'high' and 'low' resources, we recode these 'high' and 'low' resource groups at different cut-offs and re-estimate the models in Table F3. We first code 'high' resource group as those above the median (in the upper 50th percentile), and those in the 'low' resource group as those below the median. We then fit each of the six models shown in Table F3 with 'high' and 'low' resources defined as such. These models estimate the differential effect of the pandemic on access to EU policy-makers and tweet frequency between 'high' and 'low' resource group among (1) all interest groups, (2) businesses specifically, and (3) NGOs specifically. We then recode 'high' and 'low' resources at the 51st percentile, and refit the models. We estimate these models with resources defined

from the median to 90th percentile by 1 percentile increments, to capture an wide range of possible codings.

The results are presented in Figure F6. Each panel presents point estimates and 95% confidence intervals for difference-in-differences models fit to the two main outcomes for the full dataset and data from businesses and NGOs specifically. The top panels correspond to the Models (1), (2), and (3) in Table F3 respectively; the bottom panels, Models (4), (5), and (6). As the figure shows, the results from Table F3 are generally insensitive to how 'high' and 'low' resources are coded. For estimates from Table F3 that are not significantly different from zero, the estimates are also not different from zero for estimates for any coding of resources across the full range of cut-offs (top-left panel of Figure F6, and bottom row). For the estimates of the pandemic's effect on differential access to policy-makers within business interests and within NGOs, the estimates are significantly different from zero across nearly the full range of resource codings (second and third panels in Panel A), as consistent with Models (2) and (3) in Table F3. In sum, the results in Table F3 are not an artifact of how interest groups are coded as having 'high' and 'low' resources.

F.2 Sensitivity analysis of results to comparison of the richest (upper quartile) and poorest (lower quartile) interest groups

Above, we examined the sensitivity of the results concerning resources to differences codings of 'high' and 'low' resources across a wide range of cut-offs. Here, we also test whether the pandemic affected interest group access to policy-makers and social media behavior when comparing the highest-resource interest groups to the lower-resource interest groups. To do so, we subset the data to include only interest groups in the lower quartile of resources (defined as 'low') and those in the upper quartile (defined as 'high'). Using these data, we then fit the same models as included in Table F3. Results are presented in Table F4 and are effectively equivalent to those in Table F3: all point estimates are similar and are similarly statistically (in)significant to those in Table F3.

Figure F6: Sensitivity analysis of differences in the number of meetings with policy-makers and tweets by resource group

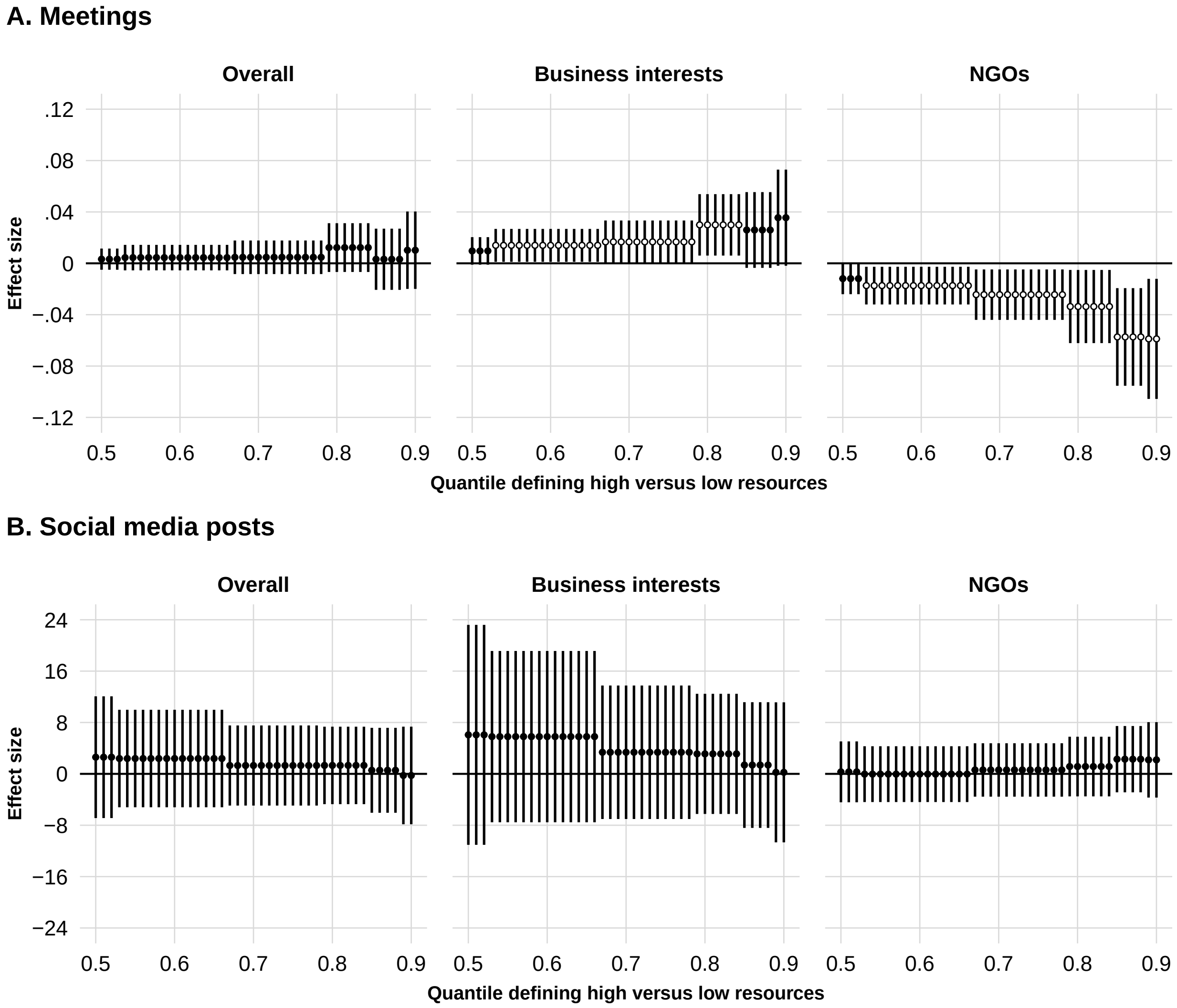

This figure shows the estimated effect of the pandemic on differences in access to meetings with policy-makers and differences in the number of tweets sent by interest groups depending on their access to resources. Each point estimate and 95% CI represents the estimated effect of the pandemic on the difference in meetings and tweets between "high" and "low" resource interest groups by defining "high" and "low" resources at different cutoffs. Points in white indicate confidence intervals that do not cross zero.

Table F4: Regression results of the differential effect of the pandemic on high-resource and low-resource interest groups (*lowest quartile* versus *upper quartile*)

	Outcome variable					
	Number of meetings			Number of tweets		
	(1)	(2)	(3)	(4)	(5)	(6)
Lock-down × Resources	0.005	0.017*	−0.024*	1.302	3.363	0.608
	(0.007)	(0.008)	(0.010)	(3.182)	(5.301)	(2.121)
Month fixed effect	✓	✓	✓	✓	✓	✓
Interest group fixed effect	✓	✓	✓	✓	✓	✓
Interest group time trends	✓	✓	✓	✓	✓	✓
Data	Businesses & NGOs	Businesses	NGOs	Businesses & NGOs	Businesses	NGOs
Observations	162,623	105,188	57,435	103,283	63,012	40,271
R^2	0.296	0.297	0.291	0.669	0.648	0.746

*p<0.05; **p<0.01; ***p<0.001. Standard errors, in parentheses, are clustered at the level of the interest group. The outcome variable is defined as the number of meetings or tweets from each interest group aggregated at the month level from January 1, 2019 to September 30, 2020.

G Sensitivity of results stratified by resources (Table 3) to alternative codings of 'high' and 'low' resources

In Table 3 of the main article, we examine whether the resources that are available to NGOs and business interests drive the results. We do so by examining the differential effect of the pandemic on access to policy-makers and social media behavior by stratifying interest groups by their available resources. In Table 3 of the main article, interest groups with 'high' resources are defined as those in the upper tercile (upper 67th percentile) of lobbying resources, and interest groups with 'low' resources are defined by those in the lower two terciles. These definitions of 'high' and 'low' resources, however, are relatively arbitrary. We thus test whether the results in Table 3 are sensitive to how high-resource and low-resource groups are coded. To do so, we recode 'high' and 'low' groups at a wide range of cut-offs—from the median thru the 90th percentile—and refit the models from Table 3 for each potential cut-off.

Estimates of the differential effect of the pandemic on access to meetings with policy-makers and social media posts among NGOs and business interests among high- and low-resource groups are presented in Figure G7. Panel A corresponds to Models (1) and (2) in Table 3 in the main article; Panel B, Models (3) and (4). The figure demonstrates that the results in Table 3 are insensitive to how 'low' and 'high' resource interest groups are coded. The left figure of Panel A shows that the pandemic caused a decrease in NGOs' access to meetings with policy-makers relative to business interests among high-resource groups, regardless of how 'high' resources is coded (all estimates are significantly different from zero). By contrast, the right figure of Panel A shows very little evidence that the pandemic caused a similar decrease in NGOs's access to meetings with policy-makers about low-resource groups, regardless of how 'low' resources is coded (all but two estimates are no significantly different from zero).

In Panel B of Figure G7, we see similarly that the results from Table 3 in the main article are relatively insensitive to the coding of resources. In both figures of Panel B, the pandemic

Figure G7: Sensitivity analysis of differences in the number of meetings with policy-makers and tweets, stratified by resource group

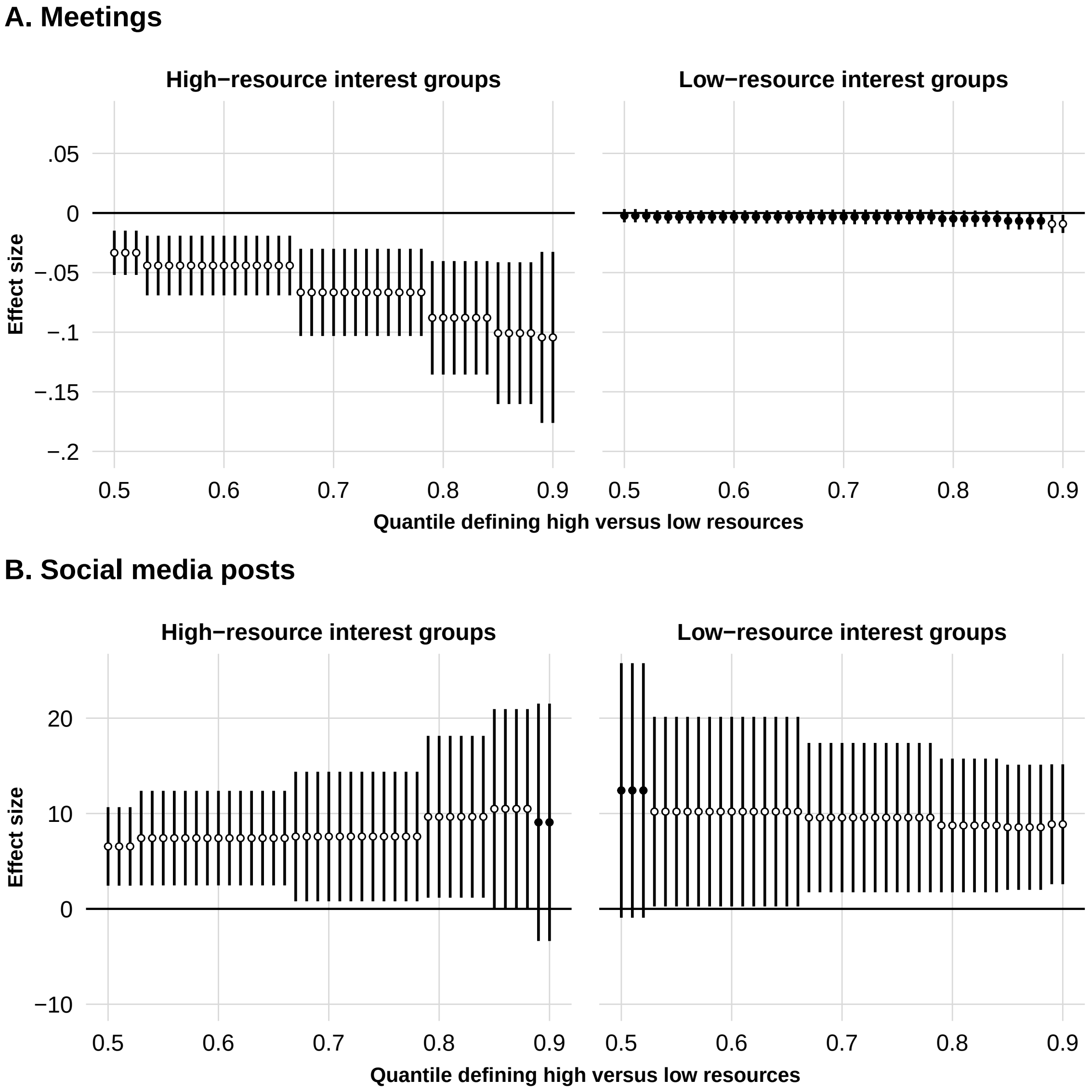

This figure shows the estimated effect of the pandemic on differences in access to policy-makers and differences in the number of tweets sent by interest groups, among NGOs relative to business interests stratified by lobbying resources. Each point estimate and 95% CI represents the estimated effect of the pandemic on differences in meetings and tweets for NGOs relative to business interests when subsetting the data at different codings of “low” and “high” resources. Points in white indicate confidence intervals that do not cross zero.

is estimated to have a caused an increase in the frequency of social media behavior by NGOs relative to businesses, both among high- and low-resource groups, regardless of how ‘high’ and ‘low’ resources are coded.

H Interest group staff size as an alternative measure of resources

In Table 3 in the main article and Table F3, we measure the resources available to interest groups by their lobbying budget, as defined in the EU Transparency Register. As a robustness, check, we also replicate these two tables using an alternative measure from the EU Transparency Register: the full-time staff size of each interest group dedicated to lobbying activities. As with the lobbying budget, we define 'high' and 'low' resource interest groups as those in the upper tercile ('high') and lower two terciles ('low') of staff sizes.

Results are presented in Table H5 and Table H6. The results in each table using staff size as an alternative measure of resource availability are substantively equivalent to those using interest groups' lobbying budget. Table H5 presents estimates of the effect of pandemic on NGO's access to policy-makers, and social media behavior relative to business interests, stratifying by resources. The results are nearly exactly equivalent to those from Table 3 in the main article. The only notable difference is that although the point estimate on the effect of the pandemic on NGOs' tweeting behavior relative to business interests in Model (3) is nearly identical, its level of statistical significance a somewhat lower ($p = 0.11$)) than when stratifying by lobbying budget. In Table H6, the estimated effects are substantively equivalent, and there are no differences in statistical significance across all six models relative to estimates using lobbying budget as a measure of resources in Table F3.

Table H5: Regression results of the effect of the COVID-19 pandemic on differences in access to policy-makers, and social media communications, among NGOs relative to business, stratified by resources (as measured by *staff size*)

	Outcome variable			
	Number of meetings		Number of tweets	
	(1)	(2)	(3)	(4)
Lock-down × NGO interest group	-0.041^{***}	-0.001	8.811	8.285^{**}
	(0.011)	(0.003)	(5.609)	(2.612)
Month fixed effect	✓	✓	✓	✓
Interest group fixed effect	✓	✓	✓	✓
Interest group time trends	✓	✓	✓	✓
Data	High resource groups	Low resource groups	High resource groups	Low resource groups
Observations	65,163	98,468	47,352	57,418
R^2	0.312	0.164	0.671	0.666

$^{*}p<0.05$; $^{**}p<0.01$; $^{***}p<0.001$. Standard errors, in parentheses, are clustered at the level of the interest group. The outcome variable is defined as the number of meetings or tweets from each interest group aggregated at the month level from January 1, 2019 to September 30, 2020.

Table H6: Regression results of the effect of the COVID-19 pandemic on access to policy-makers, and social media communications, among high-resource groups relative low-resource groups (as measured by *staff size*)

	Outcome variable					
	Number of meetings			Number of tweets		
	(1)	(2)	(3)	(4)	(5)	(6)
Lock-down × Resources	0.006	0.022^{*}	-0.019^{**}	-0.604	-1.298	-0.686
	(0.006)	(0.009)	(0.007)	(3.511)	(5.788)	(2.184)
Month fixed effect	✓	✓	✓	✓	✓	✓
Interest group fixed effect	✓	✓	✓	✓	✓	✓
Interest group time trends	✓	✓	✓	✓	✓	✓
Data	Businesses & NGOs	Businesses	NGOs	Businesses & NGOs	Businesses	NGOs
Observations	163,631	105,699	57,932	103,886	63,245	40,641
R^2	0.295	0.297	0.289	0.670	0.649	0.747

$^{*}p<0.05$; $^{**}p<0.01$; $^{***}p<0.001$. Standard errors, in parentheses, are clustered at the level of the interest group. The outcome variable is defined as the number of meetings or tweets from each interest group aggregated at the month level from January 1, 2019 to September 30, 2020.

I Regression results for the log number of meetings and tweets

In the main article, we present difference-in-differences regression models for the outcomes defined as (1) the number of meetings that each interest group has with EU policy-makers, and (2) the number of tweets sent by each interest group. As a robustness check, we also fit the main regression models to the log number of meetings and tweets.

We begin by investigating the effect of the pandemic on differences in the number of meetings that businesses and NGOs have with policy-makers and the number tweets sent by each interest group. Results for the log count of meetings and tweets (analogous to Table 1 in the main article) are presented in Table I7. The results are effectively equivalent to those in the article. Onset of the pandemic is associated with a decrease in the number meetings that NGOs had with EU policy-makers relative to business interests ($p < 0.001$). By contrast, the pandemic is associated with an increase in the frequency of tweets sent by NGOs relative to business interests ($p < 0.01$).

Table I7: Regression results of the effect of the COVID-19 pandemic on meeting access and social media behavior (log number of meetings and tweets)

	Outcome variable	
	ln Number of meetings<br>(1)	ln Number of tweets<br>(2)
Lock-down × NGO interest group	-0.008^{**}<br>(0.002)	0.066^{**}<br>(0.022)
Month fixed effect	✓	✓
Interest group fixed effect	✓	✓
Interest group time trends	✓	✓
Observations	163,631	103,886
R^2	0.288	0.864

*p<0.05; **p<0.01; ***p<0.001. Standard errors, in parentheses, are clustered at the level of the interest group. The outcome variable is defined as the log number of meetings or tweets from each interest group aggregated at the month level from January 1, 2019 to October 1, 2020.

Table I8: Regression results of the effect of the COVID-19 pandemic on meeting access and social media behavior (COVID-related meeting and tweet removed) (log number of meetings and tweets)

	Outcome variable	
	ln Number of meetings	ln Number of tweets
	(1)	(2)
Lock-down × NGO interest group	−0.003	0.029
	(0.002)	(0.022)
Month fixed effect	✓	✓
Interest group fixed effect	✓	✓
Interest group time trends	✓	✓
Observations	163,631	103,886
R^2	0.266	0.861

$^{*}p<0.05$; $^{**}p<0.01$; $^{***}p<0.001$. Standard errors, in parentheses, are clustered at the level of the interest group. The outcome variable is defined as the log number of meetings or tweets from each interest group aggregated at the month level from January 1, 2019 to October 1, 2020. Data included are those meetings and tweets that are not classified as being related to the COVID-19 pandemic.

We then fit difference-in-differences models equivalent to those in Table 2 in the main article, where the outcome is the log number of meetings and tweets for meetings and tweets that are not classified as being related to COVID-19. Results are presented in Table I8. Similar to the results presented in Table 2 of the main article, we find no strong evidence that onset of the pandemic is associated with differences in the log number of meetings that NGOs or business interests had with EU policy-makers, or the frequency of tweets sent by each class of interest group when explicitly COVID-related meetings and tweets are removed.

Finally, we stratify by the resources available to each interest group and fit models to estimate the effect of the pandemic on the log number of meetings that interest groups have with EU policy-makers and the number of tweets they send. We fit a difference-in-differences model equivalent to that used in the main article (Table 3) to the logged outcomes. Results are presented in Table I9. Compared to the analogous table in the main article (Table 3), the results are effectively equivalent. Among high-resource interest groups, the pandemic caused

Table I9: Regression results of the effect of the COVID-19 pandemic on meeting access and social media behavior, stratified by interest group resource levels (log number of meetings and tweets)

	Outcome variable			
	ln Number of meetings		ln Number of tweets	
	(1)	(2)	(3)	(4)
Lock-down × NGO interest group	-0.018^{**}	-0.002	0.115^{***}	0.043
	(0.006)	(0.002)	(0.033)	(0.029)
Month fixed effect	✓	✓	✓	✓
Interest group fixed effect	✓	✓	✓	✓
Interest group time trends	✓	✓	✓	✓
Data	High resource groups	Low resource groups	High resource groups	Low resource groups
Observations	58,390	102,963	44,773	57,955
R^2	0.305	0.153	0.858	0.863

$^{*}p<0.05$; $^{**}p<0.01$; $^{***}p<0.001$. Standard errors, in parentheses, are clustered at the level of the interest group. The outcome variable is defined as the log number of meetings or tweets from each interest group aggregated at the month level from January 1, 2019 to September 30, 2020.

a decrease in political access to policy-makers among NGOs relative to business interests (Model (1)), an effect that is not observed among low-resource interest groups (Model (2)). Finally, similar to the results presented in the main article, we find that the pandemic caused an increase in the social media frequency of NGOs relative to business interests, both among low-resource and high-resource interest groups (although not significantly so in the latter).

J Placebo intervention

Is the observed effect of the pandemic on interest group meetings with policy-makers and tweet frequency related to the month that it occurred (in March, 2020)? To test this, we fit a models equivalent those presented in Table 1 of the main article, but use a placebo intervention of one year previous, in March 2019. Results are presented in Table J10. Unlike in the main article in which we find differential effects of the pandemic on NGO access to meetings relative to business and tweet frequency, we find neither using a placebo intervention.

Table J10: Interest group type and the effect of the COVID pandemic on access to meetings with politicians and frequency of communications (placebo intervention)

	DV			
	ln Number of meetings (1)	Number of meetings (2)	ln Number of tweets (3)	Number of tweets (4)
Placebo lock-down × NGO interest group	0.003 (0.002)	0.006 (0.003)	0.008 (0.022)	−1.410 (2.886)
Observations	242,000	242,000	103,886	103,886
R^2	0.273	0.280	0.864	0.670

*p<0.05; **p<0.01; ***p<0.001. Standard errors, in parentheses, are clustered at the level of the interest group.

K Meetings results among groups with Twitter accounts

In the article, we estimate the effect of the COVID-19 pandemic on business interests' and NGOs' meetings with policy-makers, and tweet frequency among all available interest groups in the sample. However, because not every interest group has a Twitter account, the samples used to estimate the effect of the pandemic for meetings and for tweet frequency are different. In this section, we estimate the effect of the pandemic on business interests' and NGOs' meetings with policy-makers only among those interest groups that have a Twitter account (and thus are included in the tweet frequency results).

Results are presented in Table K11. The estimate from Model (1) presents the estimate of the effect of the pandemic on meetings with policy-makers among NGOs relative to business interests using all groups in the sample. This result is equivalent to that presented in Table 1 of the main article. Model (2) presents the effect only among interest groups that have a Twitter account. As the estimate shows, the result is robust to confining the sample only to interest groups with Twitter accounts, with the estimate being even greater in magnitude.

Table K11: Interest group type and the effect of the COVID pandemic on access to meetings with politicians (among groups with a Twitter account)

	Number of meetings	
	All groups	Groups w/ Twitter account
	(1)	(2)
Lock-down × NGO interest group	−0.017***	−0.025***
	(0.005)	(0.007)
Month fixed effect	✓	✓
Interest group fixed effect	✓	✓
Interest group time trends	✓	✓
Observations	163,631	103,220
R^2	0.295	0.288

*p<0.05; **p<0.01; ***p<0.001. Standard errors, in parentheses, are clustered at the level of the interest group. The outcome variable is defined as the number of meetings from each interest group aggregated at the month level, with data from January 1, 2019 to September 30, 2020.

L Differential effects of the pandemic by sub-category

In the main article, we examine the differential effect of the pandemic on business interests relative to NGOs in aggregate. In this section, we investigate this relationship across social and economic interest group *sub-categories*. We classify interest groups according to their self-described categories of interest as captured by the Transparency Register data.[2] Specifically, we aggregate these categories of interests (e.g. "Trade", "Competition", "Public health") into larger categories representing 6 overall groups: "Business and Industry", "Economic (general)", "Public health", "Environment", "Development & social affairs", and "Agriculture". Because interest groups can select multiple categories, these groupings are not mutually exclusive: an interest group might, for example, lobby in the areas of both trade and environmental regulation.

To examine differences in the effect of the pandemic on business interests and NGO among these sub-categories, we fit a series of difference-in-differences models to the subset of data from each grouping. As in the main article, the outcomes are the frequency of meetings with EU policy-makers and the frequency of social media posts on Twitter.

Results are presented in Table L12 (meetings) and Table L13 (social media posts). As Table L12 suggests, NGOs were differentially affected by the pandemic relative to business interests in terms of less access to policy-makers across all sub-categories: the differential effect of the pandemic is negative across all models, with statistically significant effects in all, but one case. The magnitude of the differential effect is largest among interest groups that concern public health, although we note that because subgroup analysis necessarily reduces the sample size for each case, comparisons across sub-categories noisy.

In Table L13, we find similarly consistent results with respect to the frequency of social media posts: in each case, the differential effect of the pandemic is positive, suggesting that the pandemic caused an increase in social media posting behavior by NGOs relative

[2]When interest groups register with the Transparency Register they are asked to select among a predefined list of 41 categories that might represent their substantive fields of interest.

to business interests across all sub-categories. The largest statistically significant effects are those among interest groups concerned with public health and the economy. We caution, however, that the relatively large standard errors that results from sub-group analysis mean that no differences across interest group sub-categories are themselves statistically significant.

Table L12: Interest group type and the effect of the COVID pandemic on access to meetings (by interest group sub-topic)

	Number of meetings						
	Business & industry	Economy (general)	Health	Environment	Research & Development	Employment & social affairs	Agriculture
Lock-down × NGO	−0.031**	−0.020**	−0.052***	−0.025***	−0.026**	−0.016	−0.032***
	(0.010)	(0.007)	(0.011)	(0.007)	(0.008)	(0.011)	(0.009)
Month fixed effect	✓	✓	✓	✓	✓	✓	✓
Interest group fixed effect	✓	✓	✓	✓	✓	✓	✓
Interest group time trends	✓	✓	✓	✓	✓	✓	✓
Observations	72,081	118,243	50,412	98,356	83,832	48,898	60,334
R^2	0.314	0.301	0.310	0.316	0.317	0.323	0.326

*p<0.05; **p<0.01; ***p<0.001. Standard errors, in parentheses, are clustered at the level of the interest group. The outcome variable is defined as the number of meetings from each interest group aggregated at the month level, with data from January 1, 2019 to September 30, 2020.

Table L13: Interest group type and the effect of the COVID pandemic on frequency of social media posts (by interest group sub-topic)

	Number of tweets						
	Business & industry	Economy (general)	Health	Environment	Research & Development	Employment & social affairs	Agriculture
Lock-down × NGO	3.281	10.124**	5.760*	5.046*	7.461	4.927	4.056
	(2.713)	(3.489)	(2.268)	(2.161)	(4.480)	(3.476)	(2.283)
Month fixed effect	✓	✓	✓	✓	✓	✓	✓
Interest group fixed effect	✓	✓	✓	✓	✓	✓	✓
Interest group time trends	✓	✓	✓	✓	✓	✓	✓
Observations	45,387	74,628	33,275	62,198	55,818	32,823	38,501
R^2	0.817	0.723	0.838	0.802	0.683	0.793	0.814

*p<0.05; **p<0.01; ***p<0.001. Standard errors, in parentheses, are clustered at the level of the interest group. The outcome variable is defined as the number of tweets from each interest group aggregated at the month level, with data from January 1, 2019 to September 30, 2020.

M Differential effects on trade unions and public authorities

The main article focuses on NGOs and business interests. However, it is useful to also examine whether system-wide shocks such a pandemic can have effects on other categories of interest groups. While trade unions represent specific economic interests, they can also be seen as representatives of broader societal interests providing them with a capacity to mobilize the wider public. Sub-national public authorities similarly reflect societal interests by representing citizens within a given geographical area at large.

To examine the differential effects of the pandemic on trade unions and sub-national public authorities, we fit difference-in-differences models, comparing these two sets of interest groups both to NGOs and business interests. Results for trade unions are presented in Table M14 and those for sub-national public authorities in Table M15. As the results show, both trade unions and sub-national public authorities are affected similarly to NGOs. The pandemic resulted in no significant differences in the number of meetings that trade unions or sub-national public authorities had with policy-makers, or the number of tweets sent, as compared to NGOs (Models 1 and 3 in Tables M14 and M15). Similar to NGOs, however, in Model (2) of Table M14 we see that trade unions witnessed a decrease in meetings with policy-makers compared to business interests ($p < 0.05$). Sub-national authorities also saw a decrease in such meetings (Model (2) of Table M15), although the difference is not statistically significant.[3] Furthermore, similar to NGOs, both trade unions and sub-national authorities increased their public communications on social media as a result of the pandemic relative to business interests (Model 4 in Tables M14 and M15). In sum, the effect of the pandemic on meetings with policy-makers and social media posts for trade unions and sub-national authorities was similar to that of NGOs. The pandemic caused an increase in meetings for business interests relative to each interest group type, and caused an in-

[3]The number of interest groups defined as sub-national authorities in in the data is small, however, and thus statistical power in these models is relatively low compared to the comparison between business interests and NGOs.

crease in social media communications among NGOs, trade unions, and sub-national public authorities relative to business interests.

Table M14: Effect of the COVID pandemic on access to meetings with politicians and number of tweets sent (comparing trade unions to NGOs and business interests)

	DV			
	ln Number of meetings		ln Number of tweets	
	(1)	(2)	(3)	(4)
Lock-down × Trade union	−0.001	−0.008*	0.035	0.100**
	(0.004)	(0.004)	(0.039)	(0.038)
Comparison category	NGOs	Business interests	NGOs	Business interests
Month fixed effect	✓	✓	✓	✓
Interest group fixed effect	✓	✓	✓	✓
Interest group time trends	✓	✓	✓	✓
Observations	75,445	123,212	50,466	73,208
R^2	0.288	0.288	0.854	0.868

*p<0.05; **p<0.01; ***p<0.001. Standard errors, in parentheses, are clustered at the level of the interest group. The outcome variables are defined as the log number of meetings, and the log number of tweets from each interest group aggregated at the month level from January 1, 2019 to October 1, 2020.

Table M15: Effect of the COVID pandemic on access to meetings with politicians and number of tweets sent (comparing sub-national public authorities to NGOs and business interests)

	DV			
	ln Number of meetings		ln Number of tweets	
	(1)	(2)	(3)	(4)
Lock-down × Public authority	−0.013	−0.020	0.113	0.177*
	(0.010)	(0.010)	(0.081)	(0.080)
Comparison category	NGOs	Business interests	NGOs	Business interests
Month fixed effect	✓	✓	✓	✓
Interest group fixed effect	✓	✓	✓	✓
Interest group time trends	✓	✓	✓	✓
Observations	59,708	107,475	41,675	64,417
R^2	0.284	0.287	0.854	0.870

*p<0.05; **p<0.01; ***p<0.001. Standard errors, in parentheses, are clustered at the level of the interest group. The outcome variables are defined as the log number of meetings, and the log number of tweets from each interest group aggregated at the month level from January 1, 2019 to October 1, 2020.

N Effects on the frequency of sharing social media posts from NGOs and business interests

In the main article, we find that the onset of the COVID pandemic increased the frequency of social media posts by NGOs relative to business interests. A useful follow-up question is whether this increase in posting also resulted in an increase among NGOs in sharing of these posts overall by ordinary users. To test this, we fit difference-in-differences models equivalent to those in the main manuscript, but where the outcome is the number of social media posts by NGOs and business interests that are "favorited" or "retweeted" (shared) by other users in a given period.

Table N16: Effect of the COVID pandemic on frequency of sharing of social media posts from NGOs and business interests

	DV			
	ln Number of favorites		ln Number of retweets	
	(1)	(2)	(3)	(4)
Lock-down × NGO interest group	0.143***	0.044	0.093**	−0.042
	(0.023)	(0.028)	(0.033)	(0.040)
Month fixed effect	✓	✓	✓	✓
Interest group fixed effect	✓	✓	✓	✓
Interest group time trends		✓		✓
Observations	103,702	103,702	103,702	103,702
R^2	0.852	0.881	0.769	0.813

*p<0.05; **p<0.01; ***p<0.001. Standard errors, in parentheses, are clustered at the level of the interest group. The outcome variable is defined as the log number of favorited and retweets of tweets from each interest group aggregated at the month level from January 1, 2019 to October 1, 2020.

Results are presented in Table N16. They show that the pandemic increased the number of favorites and retweets received by NGOs relative to business interests (Models 1 and 3, $p < 0.01$). As a robustness check, we also include interest group-level time trends to account for potential violations of the parallel trends assumption. When these trends are included, the effects of the pandemic on the number of favorites and retweets received by NGOs

relative to businesses are no longer statistically significant. We thus find suggestive, albeit not conclusive evidence, that the increase in social media posting by NGOs resulted in increases in the reach of their public communications.

O Poisson regression results

In the main article, we use OLS regression models to estimate the differential effects of the pandemic on access to meetings with policy-makers and the frequency of social media posts. Difference-in-differences models for quasi-experimental research designs typically use OLS models for both continuous and limited dependent variables (e.g. binary, count) (Angrist and Pischke, 2009, 94-99). One may be interested in estimates from generalized linear models when the outcome variable is not continuous, however. The count distributions for the number of meetings and number of tweets for each interest group aggregated across the time period of study are presented in Figures O8 and O9. The data are unsurprisingly right skewed: a large number of the over ten thousands interest groups in the data do not have meetings with policy-makers during the time period of interest, and relatively small number of interest groups send large numbers of tweets.[4]

To show estimates from a count model, we fit fixed effects poisson models analogous to the OLS models in Table 1 from the main article. Results are presented in Table O17. They show that the pandemic caused a 45% decrease in meetings for NGOs relative to business interests ($1 - e^{-0.590} = 0.45$), and a 17% increase ($e^{0.154} = 1.17$) in the frequency of tweets. We note, however, that one drawback of count models with unit-level fixed effects is that units that are observed with all zeroes across time are dropped from the sample.

[4]Models using the log of these outcomes can be found in Appendix I.

Figure O8: Count distribution of the *number of meetings* with policy-makers that each interest group had across the time period of study

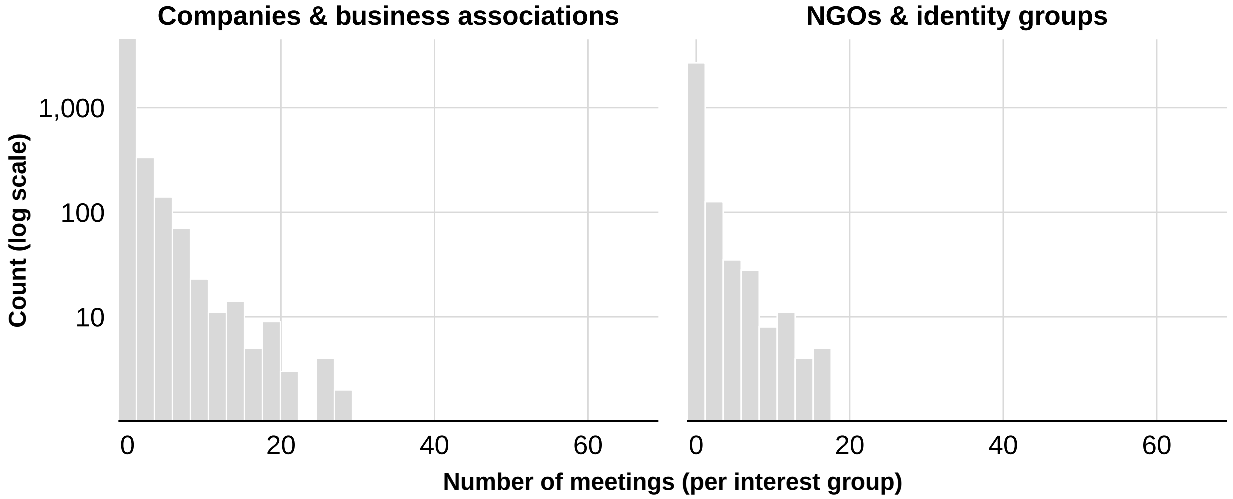

This figure shows the distribution of the number of meetings that each interest group had with policy-makers aggregated across the time period of interest (January 1, 2019, to October 1, 2020).

Figure O9: Count distribution of the *number of tweets* sent by each interest group across the time period of study

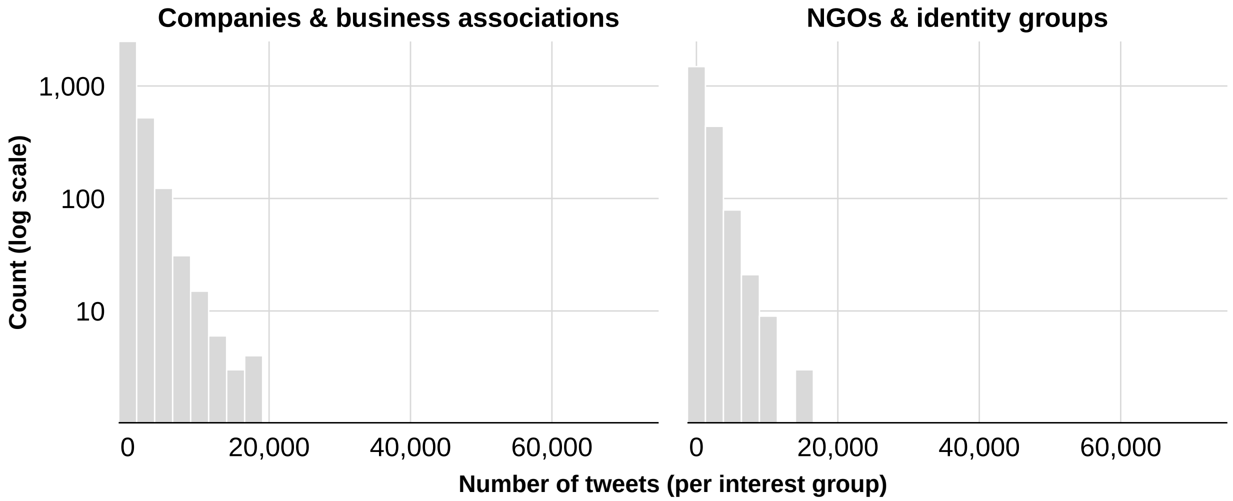

This figure shows the distribution of the number of tweets sent by each interest group aggregated across the time period of interest (January 1, 2019, to October 1, 2020).

Table O17: Poisson regression results of the effect of the COVID-19 pandemic on meeting access and social media activity

	DV	
	Number of meetings (1)	Number of tweets (2)
Lock-down × NGO interest group	−0.590*** (0.131)	0.154* (0.061)
Month fixed effect	✓	✓
Interest group fixed effect	✓	✓
Interest group time trends	✓	✓
Observations	31,857	103,578

* $p < 0.05$, ** $p < 0.01$, *** $p < 0.001$. Standard errors, in parentheses, are clustered at the level of the interest group. The outcome variable is defined as the number of meetings with policy-makers and the number of tweets sent from each interest group aggregated at the month level from January 1, 2019 to October 1, 2020.

P Differences in meetings before and during the COVID pandemic between business interest sectors

In Figure P10, we present a time series of the average number of meetings by interest groups with EU policy-makers, broken down by sector. Panel A shows that the average number of meetings with policy-markers does not appear to vary substantially by sector, with all sectors showing similar patterns over time. In Panel B, we present individual time series for each sector, relative to the average number of meetings across all sectors. Again, we do not see observe many clear between-sector patterns.

To examine potential differences more formally, we use a panel regression model that compares changes in meetings between the pre-COVID and COVID periods among business interests by sector. We note that a drawback to the sectoral data is that interest groups can indicate belonging to multiple sectors. Examining differences between groups is thus not a straightforward comparison between exclusive sets of groups. Nevertheless, regressing a set of interactions between a binary variable indicating the COVID pandemic period and each sector to which a business interest belongs can provide us with a rough idea of whether certain sectors benefited from the pandemic.

Results are presented in Table P18, where the group "Business and Industry" is used as a baseline category. The results suggest that business interests linked to public health and research and innovation may have benefited more from the pandemic in terms of access to policy-makers than did business interests associated with other categories.

Table P18: OLS regression results of the effect of the COVID-19 pandemic on meeting access by business sector

	(1)
Lock-down × economic	0.008
	(0.004)
Lock-down × public health	0.020**
	(0.007)
Lock-down × climate	−0.005
	(0.005)
Lock-down × research and innovation	0.012**
	(0.005)
Lock-down × employment and social affairs	−0.008
	(0.006)
Lock-down × agriculture	0.005
	(0.006)
Num.Obs.	154 816
R2	0.296
AIC	−14 207.6
BIC	139 987.5
Month fixed effect	✓
Interest group fixed effect	✓
Interest group time trends	✓

* $p < 0.05$, ** $p < 0.01$, *** $p < 0.001$

Figure P10: Number of meetings of interest groups with EU policy-makers by sector

A. Number of meetings per business interest category

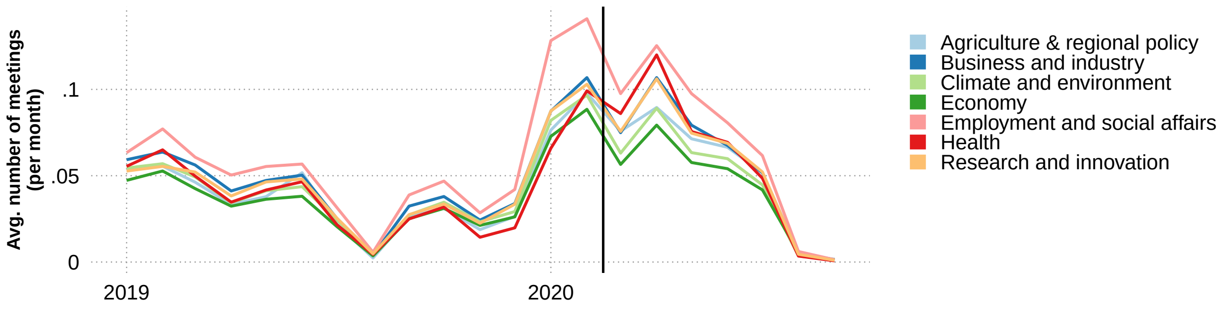

B. Difference in number of meetings from average

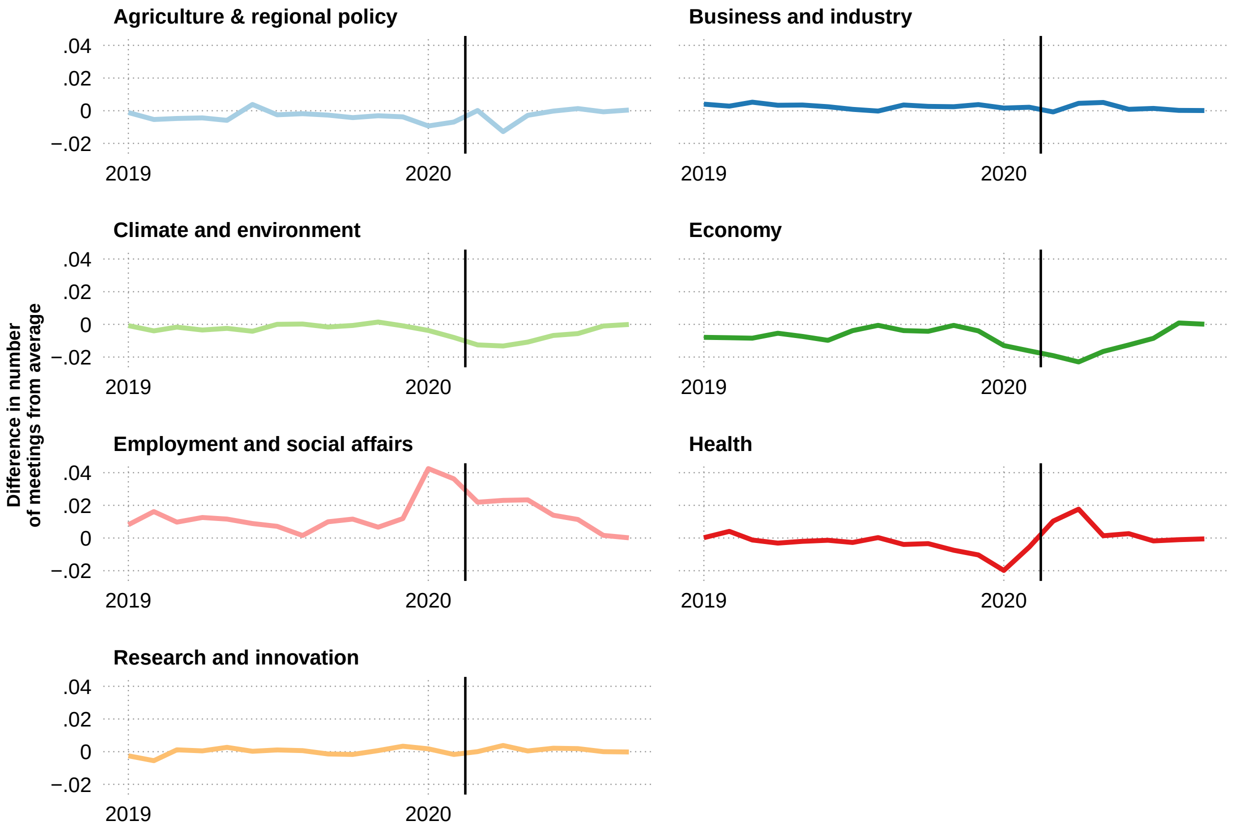

Q Interest groups that saw largest increase in meetings during the pandemic

To provide qualitative background for understanding the interest groups that may have benefited from the pandemic, we present in Table Q19 a list of the 50 interest groups that saw the largest increase in their average number of meetings with policy-makers per month between the pre-COVID and COVID periods.

R Differential effects of the pandemic by companies and other business interest groups

In the main article, we examine the overall effects of NGOs relative to business interests. As shown in Table B1, business interests are defined by a variety of sub-categories. Here, we examine the effects of the pandemic on differences separately for NGOs compared to "Companies & groups", and NGOs compared to interest groups that are defined by the other categories ("Professional consultancies", "Self-employed consultants", "Law firms", and "Trade and business associations"). Results for the effect of the pandemic on NGOs' meetings with policy-makers and the frequency of communication on social media relative to business associations and companies are shown in Table R20. As the table shows, NGOs have fewer meetings as a result of the pandemic relative to both business associations and companies (Models 1 and 2), and send more tweets than business associations and companies (Models 3 and 4), although only significantly so for social media communications relative to companies.

Table Q19: Top 50 interest groups that increased their average number of meetings with policy-makers in the COVID period

	**Organization**
1	European Federation of Pharmaceutical Industries and Associations (EFPIA)
2	MedTech Europe (MTE)
3	Vaccines Europe (VE)
4	Association of the European Self-Care Industry (AESGP)
5	BUSINESSEUROPE
6	European Coordination Committee of the Radiological, Electromedical and healthcare IT Industry (COCIR)
7	MEDICINES FOR EUROPE (-)
8	CEEP - European Centre of Employers and Enterprises providing Public Services and Services of General Interest (CEEP)
9	European Confederation of Pharmaceutical Entrepreneurs (EUCOPE)
10	American Chamber of Commerce to the European Union (AmCham EU)
11	Norwegian Refugee Council Europe (NRC Europe)
12	ENEL SpA
13	AGE Platform Europe (AGE)
14	Médecins Sans Frontières International (MSF International)
15	Climate Action Network Europe (CAN Europe)
16	European Chemical Industry Council (Cefic)
17	World Economic Forum (WEF)
18	European Environmental Bureau (EEB)
19	Hydrogen Europe (HE)
20	SMEunited aisbl (SMEunited)
21	Volvo AB (Volvo Group)
22	AIR LIQUIDE (AIR LIQUIDE)
23	AMADEUS IT GROUP S.A.
24	International Federation of Organic Agriculture Movements EU Regional Group (IFOAM EU Group)
25	AeroSpace and Defence Industries Association of Europe (ASD)
26	European agri-cooperatives (COGECA)
27	Hope and Homes for Children (HHC)
28	SDG Watch Europe
29	Associazione delle Imprese del farmaco (FARMINDUSTRIA)
30	L'Oréal
31	Voluntary Organisations in Cooperation in Emergencies (VOICE asbl)
32	Siemens AG (SAG)
33	Airbus
34	Green 10
35	Transport and Environment (European Federation for Transport and Environment) (T&E)
36	International Trademark Association (INTA)
37	The Council of European Professional Informatics Societies (CEPIS)
38	Euromontana (Euromontana)
39	Siemens Healthineers AG (SHS)
40	WindEurope
41	ActionAid (AAI)
42	eu travel tech
43	Koninklijke Philips (Philips)
44	EIT Health
45	ArcelorMittal (AM)
46	Association for Financial Markets in Europe (AFME)
47	The European Region of the International Lesbian, Gay, Bisexual, Trans and Intersex Association (ILGA-Europe)
48	European Atomic Forum (FORATOM)
49	BASF SE
50	European Ageing Network (E.A.N.)

Table R20: Companies and remaining business interests and the effect of the COVID pandemic on access to meetings with politicians and frequency of communications

	Number of meetings		Number of tweets	
	(1)	(2)	(3)	(4)
Lock-down × NGO interest group	−0.019**	−0.015*	16.688**	1.252
	(0.006)	(0.006)	(5.620)	(1.424)
Data	Companies	Remaining business interests	Companies	Remaining business interests
Observations	120 197	101 366	71 076	74 715
R2	0.296	0.292	0.655	0.762

* $p < 0.05$, ** $p < 0.01$

References

Angrist, Joshua D. and Jörn-Steffen Pischke. 2009. *Mostly Harmless Econometrics: An Empiricist's Companion*. Princeton, NJ: Princeton University Press.

Blavatnik School of Government. 2021. "COVID-19 Government Response Tracker." Blavatnik School of Government, Univeristy of Oxford, https://www.bsg.ox.ac.uk/research/research-projects/coronavirus-government-response-tracker.

Cunningham, Scott. 2021. *Causal Inference: The Mixtape*. New Haven, CT: Yale University Press.
